# Supplementary material for: Protocol of a parallel group Randomized Control Trial (RCT) for Mobile-assisted Medication Adherence Support (Ma-MAS) intervention among Tuberculosis patients
Source: PLoS One. 2021 Dec 31;16(12):e0261758. doi: 10.1371/journal.pone.0261758 (PMC8719740; doi:10.1371/journal.pone.0261758)
Supplement: S2 Checklist — (PDF) [file pone.0261758.s002.pdf]

English Version checklist for phone calls intervention

(To be completed by a health professional at sub-city clinic when participant get a phone call)

**Identification**
Sub-city Name: _________________<br>Health facility Name: ________________<br>Participant registration code: ________________<br>Participant mobile phone number: ________________

Weekly phone calls intervention checklist form

Questions to be asked of the participant by a health professional:

Please answer the following questions by saying 'Yes' or 'No" based on the intervention you are currently receiving.

			**Ask the patient the following questions when you call**			
**Weeks**	**Date (DD/MM/YY)**	Are the patient answered for the phone calls (minimum of 3 repetitive calls)	Good morning, do you taking your daily medication?	Are you reading and following the SMS text message instructions?	Do you have any health problems or side-effects you want to discuss?	Do you have a question regarding your treatment?
Week 1						
Week 2						
Week 3						
Week 4						
Week 5						
Week 6						
Week 7						
Week 8						
