# Supplementary material for: Protocol of a parallel group Randomized Control Trial (RCT) for Mobile-assisted Medication Adherence Support (Ma-MAS) intervention among Tuberculosis patients
Source: PLoS One. 2021 Dec 31;16(12):e0261758. doi: 10.1371/journal.pone.0261758 (PMC8719740; doi:10.1371/journal.pone.0261758)
Supplement: S1 Table — (DOCX) [file pone.0261758.s003.docx]

S1Table: SMS text messaging interventions for TB treatment support

| First author (year) | Country | Sample Size | Study Design | Population | Intervention | Intervention duration | One way or Two way | Text messaging frequency | Additional reminder | Control | Outcome | Major finding |
| --- | --- | --- | --- | --- | --- | --- | --- | --- | --- | --- | --- | --- |
| Ali et al. (2019)  (1) | Sudan | 148 | A Parallel group Non-randomized trial | Newly diagnosed Pulmonary TB patients age 15 years and above | Patients who had access to mobile phone received an SMS text message in addition to standard care. | 6 months | Two-way | In every 48 hours in the first two months and weekly basis from two months to end of treatment | Patients also received at least one telephone call during the first week of TB treatment. Text messaging in case the patient interrupt treatment. | Patients who do not had access to mobile phone did not received SMS text or telephone call | Treatment default and treatment cure | The intervention group had lower treatment default compared to the control group (6.8% Vs 10.8%). This difference was not statistically significant P value > 0.05 (OR=1.67, 95%CI: 0.52, 5.37). The intervention group had a statistically higher TB cure rate compared with the control group P-value 0.020 (OR=2.47, 95%CI: 1.13, 5.43) |
| Alotaibi (2019)  (2) | Jeddah | 216 | A parallel group randomized control trial (RCT) | Pulmonary TB patients age 13 years and above | Patients received an SMS text reminder in addition to standard care. | 2 months | One-way | Daily | No | Patients did not received SMS text reminders except standard care. | Treatment adherence and smear-conversion rates | There was a statistically significant higher in smear conversion rate in the intervention compared with the control group on smear conversion P-value <0.001. The proportion of smear-negative at end of two months of treatment was 72.6% in the intervention group and 52.4% in the control group. A statistically significant higher self-report adherence to treatment also observed among the intervention group (P value<0.001) |
| Bediang et al. (2018) & (2014) (3, 4) | Cameroon | 279 | A parallel group RCT | Pulmonary TB patients age 18 years and above | Patients received an SMS text reminder in addition to standard care. | 6 months | one-way | Daily | Weekly motivational text | Patients did not received SMS text reminders except standard care. | Treatment cure, self-report adherence, clinic attendance of appointments | At five months treatment success was 81% in the intervention group and 74.6% in the control group P value 0.203; (OR=1.45, 95%CI: 0.81, 2.56). At six months the treatment cured rate was 63.5% in the intervention group and 62% in the control group. At six months drop out was not a statistical difference between two groups; 34.3% in the intervention group and 32.4% in the control group. |
| First author (year) | **Country** | **Sample Size** | **Study Design** | **Population** | **Intervention** | **Intervention duration** | **One way or Two way** | **Text messaging frequency** | **Additional reminder** | **Control** | **Outcome** | **Major finding** |
| Fang et al. (2017)  (5) | China | 350 | Cluster RCT | Pulmonary TB patients age 15 years and above | Patients received SMS text reminder in addition to standard care. | 6 months | one-way | Daily | No | Patients did not received SMS text reminders except standard care. | Treatment completion, Missed dose, interrupted treatment, a re-examination of sputum after 2-, 5-and 6-months of treatment | The treatment completion rate was statistically significantly higher in the intervention group (96.25%) compared with the control group (86.84%)(P-value<0.002). Statistically significant lower missed dose and treatment interruption was found; the missed dose was 18.75% in the intervention group and 45.79% in the control group and Interruption treatment rate was 17.50% in the intervention group and 32.63% in the control group (p value <0.001). There was also a statistically significant difference in the re-examination of sputum after 2 months (96.88% Vs 87.89%) and 6 months of treatment (88.13% Vs 69.47%) (p value<0.001) |
| Farooqi et al. (2017)  (6) | Pakistan | 148 | A parallel group RCT | Newly diagnosed pulmonary and extra pulmonary TB patients | Patients received a daily SMS text reminder in addition to standard care. | 2 months | one-way | Daily | No | Patients did not received SMS text reminders except standard care. | Treatment default | There was no statistically significant difference between intervention and control group on treatment default rate; 4.1% in the intervention group and 5.4% in the control group (P value >0.05). |
| Hermans et al. (2017)  (7) | Uganda | 582 | A quasi-experimental | TB-HIV 18 years and above | Patients received SMS text in addition to standard care. | 2 months | Two-way | SMS reminder in every five days and educational quizzes text in every 2 weeks | No | Patients did not received any SMS text except standard care. | Loss to follow up | There was no statistical difference in the risk of loss to follow up in the intervention group compared (0.5%) to the control group (2%) (RR 0.27, 95% CI 0.03–2.07; P=0.22). |

| First author (year) | Country | Sample Size | Study Design | Population | Intervention | Intervention duration | One way or Two way | Text messaging frequency | Additional reminder | Control | Outcome | Major finding |  |  |
| --- | --- | --- | --- | --- | --- | --- | --- | --- | --- | --- | --- | --- | --- | --- |
| Husler et al. (2005)  (8) | South Africa | 221 | A quasi-experimental | All type of Active TB patients | Patients received SMS text reminder in addition to standard care. | 6 -8 months | One-way | Daily | No | Patients did not received SMS text reminders except standard care. | TB cure rates and treatment completion rates | There was no statistical significance difference on TB cure and treatment completion rates between the intervention and control groups; cure rate (62.35% Vs 66.4%) completion rate (10.59% Vs 3%) and treatment success rate (72.94% Vs 69.4) |  |  |
| Iribarren et al. (2013)  (9) | Argentina | 37 | Mixed method design including A parallel group RCT | All type of Active TB 18 years and above | Patients received SMS text reminder in addition to standard care. | 2 months | Two-way | Daily | Weekly motivational text. Confirmation of message receipt and a reminder sent to the patient if they did not send | Patients did not received SMS text reminders except standard care. | self-report treatment adherence  microscopy test result from positive to negative, treatment outcome | There was no statistical significance difference self-reported adherence among intervention group =77% Self-reported adherence compared with control group =53% calendar-based self-report adherence.  A similar finding obtained for sputum smear or culture between intervention and control group. |  |  |
| Johnston et al. (2018) Kop et al. (2014) (10, 11) | Canada | 358 | A parallel-group RCT | Adult Latent TB patients age 19 years and above | Patients received SMS text reminder in addition to standard care. | 12 months | Two-way | Weekly | Participants received second SMS text about their health status if they don't replay to the first SMS text. The participant who had health issues to discuss and who do not respond for two SMS text message were get a Phone call | Patients did not received SMS text reminders except standard care. | treatment completion | There was no statistically significant difference in the proportion of participants completing LTBI therapy in the intervention and control groups (79.4% Vs 81.9%, RR 0.97, 95% CI 0.88–1.07; p=0.550). |  |  |
| Kumboyono (2017)  (12) | Indonesia | 90 | A quasi-experimental | All types of TB patients | Patients received SMS text reminder in addition to standard care. | NR | NR | NR | NO | Patients did not received SMS text reminder except standard care. | Treatment compliance | Treatment compliance among the intervention group was 93.3% compared with a control group 80% which was not statistically significant (P-value >0.059). |  |  |

| First author (year) | Country | Sample Size | Study Design | Population | Intervention | Intervention duration | One way or Two way | Text messaging frequency | Additional reminder | Control | Outcome | Major finding |
| --- | --- | --- | --- | --- | --- | --- | --- | --- | --- | --- | --- | --- |
| Liu et al. (2015)  (13) | China | 4292 | Cluster RCT | Newly diagnosed TB patients | Arm1-Two-way SMS text reminders Arm2-medication monitor Arm3-SMS text reminders plus medication monitor | 6 months | Two-way | Daily | Up to 3 SMS reminders, patients expected to replay and reminders stop once the patient responds. SMS reminder sent 4, 3, 2, and1 d before the scheduled monthly follow-up visit | Self-administered therapy, family supervision, or DOT | Poor adherence-at least 20% dose missed & Patient lost to follow up | The proportion of poor adherence was: Arm1= 27.3%, Arm2=17.00%, Arm3=13.90% Control=29.90% SMS reminders only was not a statistically significant difference in adherence, but medication monitors and the combination of SMS reminders and medication monitors statistically significantly improved medication adherence. However, SMS reminders have an effect on lost to follow up. |
| Mohammed et al. (2016) (14) | Pakistan | 2207 | A parallel group RCT | Newly diagnosed TB patients | Patients received SMS text reminder in addition to standard care. | 6 months | Two-way | Daily | If the patient did not respond within two hours, a second reminder was sent. A third and final reminder for the day was sent after two additional hours of non-response. Members of the study team phoned participants who did not respond for seven days | Patients did not received SMS text reminder except standard care (DOT) | Treatment success-cured (i.e. sputum smear or culture negative in the last month) or treatment completion, death, & self-reported adherence | There was no significance difference on treatment success ARM1=83% and Control=83%, default; ARM1=10% and control=9%, Died; ARM1=2% and control= 2%, treatment failure; ARM1=2% and control=3% Additionally, there was no significant difference in self-report medication differences. |
| Yoeli et al. (2019) (15) | Kenya | 1104 | A parallelgroup  RCT | All types of TB patients | Patients received SMS text reminder in addition to standard care. | NR | Two-way | Daily | If the patient did not respond the first and second SMS text additional SMS text reminder and phone call was made | Patients did not received SMS text reminder except standard care | Treatment outcome | Unsuccessful treatment outcomes occurred in 70 patients (13.1%) in the control group and 24 patients (4.2%) in the intervention group (P<0.001). |
| Belknap (2017) (16) | US, Spain, Hong Kong, and South Africa | 964 | Individual RCT | Latent TB patients | Arm1-Weekly SMS  Reminders plus self-administered therapy  Arm2- self-administered therapy | 4 months | One -way | Weekly | No | Patients did not received SMS text reminder except standard care (DOT-monthly) | Treatment completion -11 or more doses within 16 weeks | Self-administered therapy and combination of self-administered therapy and SMS reminders not statistically lower compared to DOT. Arm1=76.4%, Arm2=74.0% Control=87.2% |

References

1. Ali AOA, Prins MH. Mobile health to improve adherence to tuberculosis treatment in Khartoum state, Sudan. Journal of public health in Africa. 2019;10(2):1101.

2. Alotaibi NN. Effect of Using Mobile Phone Messaging Reminders in Improving Adherence to Treatment of Pulmonary Tuberculosis Patients in Jeddah, During 2016-2017: A Randomized Control Study. 2019.

3. Bediang G, Stoll B, Elia N, Abena JL, Geissbuhler A. SMS reminders to improve adherence and cure of tuberculosis patients in Cameroon (TB-SMS Cameroon): a randomised controlled trial. BMC public health. 2018;18(1):583.

4. Bediang G, Stoll B, Elia N, Abena JL, Nolna D, Chastonay P, et al. SMS reminders to improve the tuberculosis cure rate in developing countries (TB-SMS Cameroon): A protocol of a randomised control study. Trials. 2014;15(1).

5. Fang XH, Guan SY, Tang L, Tao FB, Zou Z, Wang JX, et al. Effect of Short Message Service on Management of Pulmonary Tuberculosis Patients in Anhui Province, China: A Prospective, Randomized, Controlled Study. Medical science monitor : international medical journal of experimental and clinical research. 2017;23:2465-9.

6. Farooqi RJ, Ashraf S, Zaman M. THE ROLE OF MOBILE SMS-REMINDERS IN IMPROVING DRUGS COMPLIANCE IN PATIENTS RECEIVING ANTI-TB TREATMENT FROM DOTS PROGRAM.(short message service)(tuberculosis)(Report). Journal of Postgraduate Medical Institute. 2017;31(2):156.

7. Hermans SM, Elbireer S, Tibakabikoba H, Hoefman BJ, Manabe YC. Text messaging to decrease tuberculosis treatment attrition in TB-HIV coinfection in Uganda. Patient preference and adherence. 2017;11:1479-87.

8. Hüsler J. Evaluation of the on Cue compliance service pilot: Testing the use of SMS reminders in the treatment of tuberculosis in Cape Town, South Africa. 2005.

9. Iribarren SJ, Cato K, Falzon L, Stone PW. What is the economic evidence for mHealth? A systematic review of economic evaluations of mHealth solutions. PloS one. 2017;12(2).

10. Johnston JC, van der Kop ML, Smillie K, Ogilvie G, Marra F, Sadatsafavi M, et al. The effect of text messaging on latent tuberculosis treatment adherence: a randomised controlled trial. The European respiratory journal. 2018;51(2).

11. van der Kop ML, Memetovic J, Patel A, Marra F, Sadatsafavi M, Hajek J, et al. The effect of weekly text-message communication on treatment completion among patients with latent tuberculosis infection: study protocol for a randomised controlled trial (WelTel LTBI). 2014;4(4):e004362.

12. Kumboyono. Short message service as an alternative in the drug consumption evaluation of persons with tuberculosis in Malang, Indonesia. Japan journal of nursing science : JJNS. 2017;14(2):112-6.

13. Liu X, Lewis JJ, Zhang H, Lu W, Zhang S, Zheng G, et al. Effectiveness of Electronic Reminders to Improve Medication Adherence in Tuberculosis Patients: A Cluster-Randomised Trial. PLoS medicine. 2015;12(9):e1001876.

14. Mohammed S, Glennerster R, Khan AJ. Impact of a Daily SMS Medication Reminder System on Tuberculosis Treatment Outcomes: A Randomized Controlled Trial. PloS one. 2016;11(11):e0162944.

15. Yoeli E, Rathauser J, Bhanot SP, Kimenye MK, Mailu E, Masini E, et al. Digital Health Support in Treatment for Tuberculosis. The New England journal of medicine. 2019;381(10):986-7.

16. Belknap R, Holland D, Feng PJ, Millet JP, Caylà JA, Martinson NA, et al. Self-administered Versus Directly Observed Once-Weekly Isoniazid and Rifapentine Treatment of Latent Tuberculosis Infection: A Randomized Trial. Annals of internal medicine. 2017;167(10):689-97.

17. Iribarren S, Beck S, Pearce PF, Chirico C, Etchevarria M, Cardinale D, et al. TextTB: A Mixed Method Pilot Study Evaluating Acceptance, Feasibility, and Exploring Initial Efficacy of a Text Messaging Intervention to Support TB Treatment Adherence. Tuberculosis research and treatment. 2013;2013:349394.

18. Albino S, Tabb KM, Requena D, Egoavil M, Pineros-Leano MF, Zunt JR, et al. Perceptions and acceptability of short message services technology to improve treatment adherence amongst tuberculosis patients in Peru: a Focus Group Study. PloS one. 2014;9(5):e95770.

19. Nhavoto JA, Grönlund Å, Klein GO. Mobile health treatment support intervention for HIV and tuberculosis in Mozambique: Perspectives of patients and healthcare workers. PloS one. 2017;12(4):e0176051.

20. Hoffman JA, Cunningham JR, Suleh AJ, Sundsmo A, Dekker D, Vago F, et al. Mobile direct observation treatment for tuberculosis patients: a technical feasibility pilot using mobile phones in Nairobi, Kenya. American journal of preventive medicine. 2010;39(1):78-80.

21. de Sumari-de Boer IM, van den Boogaard J, Ngowi KM, Semvua HH, Kiwango KW, Aarnoutse RE, et al. Feasibility of Real Time Medication Monitoring Among HIV Infected and TB Patients in a Resource-Limited Setting. AIDS and behavior. 2016;20(5):1097-107.

22. Mohammed S, Siddiqi O, Ali O, Habib A, Haqqi F, Kausar M, et al. User engagement with and attitudes towards an interactive SMS reminder system for patients with tuberculosis. 2012;18(7):404-8.
