# Supplementary material for: Protocol of a parallel group Randomized Control Trial (RCT) for Mobile-assisted Medication Adherence Support (Ma-MAS) intervention among Tuberculosis patients
Source: PLoS One. 2021 Dec 31;16(12):e0261758. doi: 10.1371/journal.pone.0261758 (PMC8719740; doi:10.1371/journal.pone.0261758)
Supplement: S2 Table — (DOCX) [file pone.0261758.s004.docx]

S2 Table: SMS interventions for TB: Acceptability and Feasibility study design

| Table 4: SMS interventions for TB: Acceptability and Feasibility study design | | | | | | | |
| --- | --- | --- | --- | --- | --- | --- | --- |
| Source | **Country** | **Sample size** | **Study design** | **Population** | **Intervention** | **Outcome measure** | **Major findings** |
| Iribarren, 2013 (17) | Argentina | 37 | Quantitative | Active TB | Two-way SMS | Feasibility, acceptability for treatment adherence | SMS intervention found as feasible as it has high access to mobile phones, patient familiarity with texting, and low refusal. SMS also found as acceptable as patients had a feeling of care, supported and responsible. |
| Albino 2014 (18) | Peru | 4 FGD | Qualitative | Active TB | One way-SMS | Perception and acceptability for treatment adherence | Patients have positive perception and accept mobile based SMS for improving medication adherence through transmit motivational text and simple reminders. |
| Nhavoto 2017(19) | Mozambique | 140 patients  40 Health professional | Qualitative | Active TB &  Healthcare professional | Two-way SMS | Usefulness, perceived benefits, ease of use, Perception satisfaction, risks of the SMS | SMS was found as useful and reliable because majorly reduce failure to collect medication and avoiding a missing appointment. Patients were confident on SMS and two way SMS (questions and appropriate answer) likable features. Unintentional disclosure of health status was the risk in case patient use shared mobile phone. |
| Sumari-de 2016 (21) | Tanzania | 10 Patients | Quantitative and qualitative | Active TB | SMS reminder when real-time medication monitoring | Process measure, adherence, perception | Patients reported that they satisfied with SMS reminder but few reported that SMS reminder may be seen by others. However, patients were disturbed by incorrect SMS reminders. |
| Mohammed 2012 (22) | Pakistan | 30 patients | Qualitative | Active TB | Two-way | Acceptability and engagement | Interactive two-way SMS reminders were acceptable by patients with Tuberculosis to support medication adherence. However, the average response rate for SMS was 57% (62% during the first message of the week and 49% during the last ten days of messaging) |
