# Supplementary material for: Protocol of a parallel group Randomized Control Trial (RCT) for Mobile-assisted Medication Adherence Support (Ma-MAS) intervention among Tuberculosis patients
Source: PLoS One. 2021 Dec 31;16(12):e0261758. doi: 10.1371/journal.pone.0261758 (PMC8719740; doi:10.1371/journal.pone.0261758)
Supplement: S1 File — (PDF) [file pone.0261758.s005.pdf]

Participant Information Sheet/Consent Form
Intervention and post-intervention Participants

Interventional Study - Adult providing own consent

**Title**	Mobile-assisted Medication Adherence Support (Ma-MAS) intervention for Tuberculosis Patients: Adoption of Intention, Effectiveness, and Experiences in Addis Ababa, Ethiopia.
**Sub-title**	Mobile-assisted Medication Adherence Support Intervention Effectiveness: Randomized control Trial (RCT)
**Short Title**	Ma-MAS intervention Effectiveness: RCT
**Protocol Number**	PACTR202002831201865
**Project Sponsor**	Flinders University, South Australia
**Coordinating Principal Investigator/ Principal Investigator**	Professor Paul Arbon
**Associate Investigator(s)**	Zekariyas Sahile Nezenega; Dr Lua Perima-Lewis Professor Anthony Maeder
**Location**	Government Health Facility, Addis Ababa, Ethiopia

Part 1 What does my participation involve?

1 Introduction

You are invited to take part in this research project because you have been following your anti-Tuberculosis treatment at this health facility. The research project is testing a new behavioural intervention for Tuberculosis medications adherence. The new behavioural intervention is called Mobile-assisted Medication Adherence Support (Ma-MAS).

This Participant Information Sheet/Consent Form tells you about the research project. It explains the tests and behavioural intervention involved. Knowing what is involved will help you decide if you want to take part in the research.

Please read this information carefully. Ask questions about anything that you don't understand or want to know more about. Before deciding whether or not to take part, you might want to talk about it with a relative, friend or your doctor or Health care provider.

Participation in this research is voluntary. If you don't wish to take part, you don't have to. You will receive the best possible healthcare whether or not you take part in this study.

If you decide you want to take part in the research project, you will be asked to sign the consent section. By signing the consent form you are telling us that you:
• Understand what you have read
• Consent to take part in the research project
• Consent to have the tests and behavioural intervention that are described
• Consent to the use of your personal and health information as described

You will be given a copy of this Participant Information and Consent Form to keep.

2 What is the purpose of this research?

The research aims to measure the effectiveness of a mobile-assisted medication adherence support intervention on patients' treatment adherence. This is an experimental behavioural intervention that is given through mobile SMS texts and phone calls in Ethiopia. The mobile-assisted medication adherence intervention has been developed systematically and tested for feasibility, and acceptability in previous stages of this research project (see Figure 1).

At present, there is a lack of well-investigated knowledge on how behavioural interventions using mobile SMS and phone calls could be effective for Tuberculosis medication adherence. This research, therefore, would provide evidence that would help healthcare managers and policymakers to develop strategies to improve Tuberculosis medication adherence and treatment outcomes. The research findings will also be used as a basis for future research and used for educational purposes.

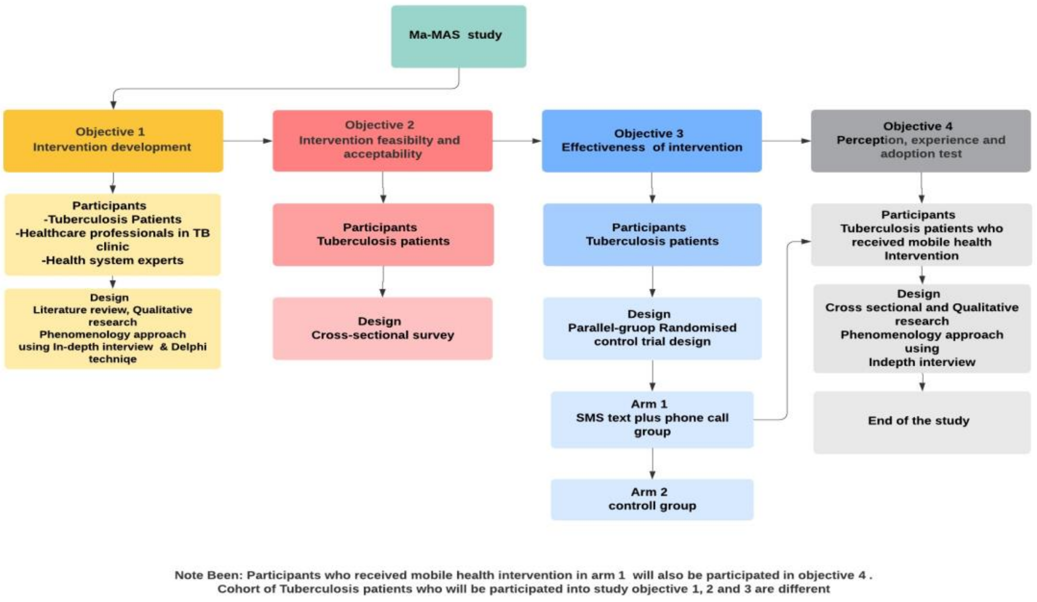

Figure 1: The flow of the study objectives

This research is conducted by collaboration between Flinders University and Addis Ababa Health Bureau. The research results will be used in the study programme for the Doctor of Philosophy degree being undertaken by the candidate Mr Zekariyas Sahile at Flinders University, Australia.

3 What does participation in this research involve?

To be involved in this research, you must agree voluntarily before any assessment or interview is made, by signing the consent form. You cannot be involved in this study unless you fulfil all the following conditions:

- participants are adult (age 18 years and above)
- participants will be following their anti-Tuberculosis treatment for six months
- participants are at the intensive phase of treatment during the screening
- participants can read and understand the national official language (Amharic)

- Participants have their own mobile phone, however, participants who don't have their own mobile phone may be included if they have a trusted family member's mobile phone in the household with a collaborative agreement

However, you will not be involved if you are deemed not to have the capacity to decide reasonably to participate, or if you are enrolled in other behavioural interventions.

You will be participating in a randomised controlled research project. Sometimes we do not know which behavioural intervention is best for treating a condition. To find out, we need to compare different behavioural interventions. We put people into groups and give each group a different behavioural intervention which may use mobile SMS text messages and phone calls (see Figure 2). The results are compared to see if one intervention is better. To try to make sure the groups are unbiased, each participant is put into a group by chance (random). If you agree to participate in the study you will be assigned into one of two groups.

1. Group one participants will receive daily SMS text messages and weekly phone calls for daily medication intake and medication refill visit reminders
2. Group two participants will receive the standard treatment as group one but not receive an additional intervention on a mobile phone.

You will know which of the group you have to be assigned, but you cannot choose yourself.

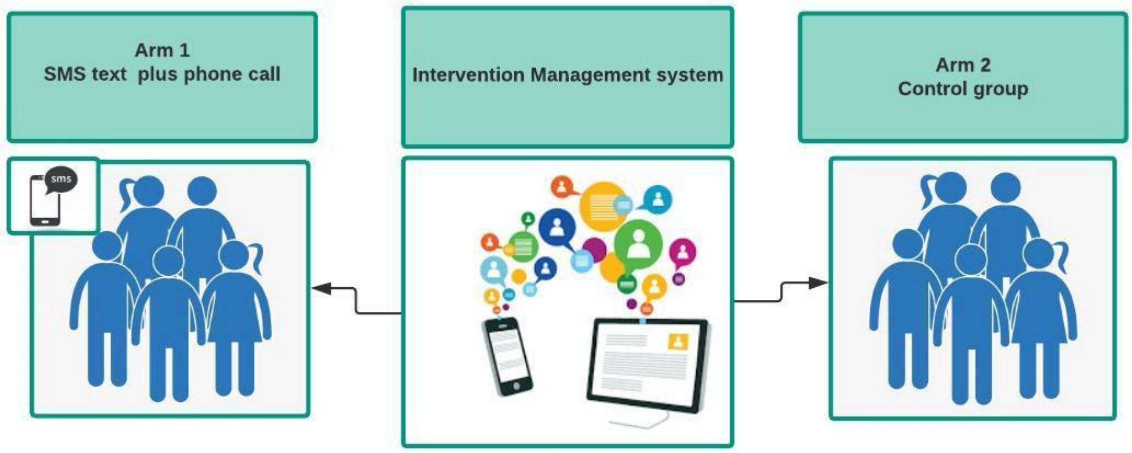

Figure 2: Mobile-assisted Medication Adherence Support (Ma-MAS) intervention

You are asked to participate in this research because the information found from this study can help the improvement of Tuberculosis treatment services. Moreover, your participation is confirmed by the probability sampling technique which provides an equal chance of selection. You have a 50% chance to participate in one of the interventions group and a 50% chance of being in the control group. If you are assigned into one of the intervention group, you will receive a daily SMS text and weekly phone calls reminder of medication intake and refill visit reminders for 2 months while you are on anti-Tuberculosis treatment during the continuation phase of treatment. If you have a local doctor or health care provider, we strongly recommend that you inform them of your participation in this research project.

This research project has been designed to make sure the researchers interpret the results fairly and appropriately and avoid study researchers or participants jumping to conclusions. If you agree to participate in this research, the following procedures will be undertaken:

- One or more members of the research team will interview you about a few socio-demographic, health service, and disease-related questions at the beginning of the study. This interview takes about 20 minutes and will be conducted in your usual clinic session.

- At subsequent clinic visits for your medication refills, the data collector will assess your medications adherence level by asking you questions about your medication usage and by taking a sample of urine for testing at a random point during months 3 to 4 of your treatment. The maximum amount of time you will spend in contact with data collectors for this purpose is 10 minutes for each time of assessment. All adherence assessments will be made immediately after you have finished your regular treatment services.

- After 2 months for the intervention and follow up period, If you have participated in one of the intervention groups listed above, you will also be asked to participate in another study and you may be a part of an in-depth interview and survey interview to assess the adoption, perceptions and experiences of mobile-assisted medication adherence support. The maximum time needed for this one-time In-depth interview and survey interview is 60 and 15 minutes, respectively.

The information obtained from this study will be used for research proposes only to inform possible solutions to improve future anti-Tuberculosis medication adherence. Your identifiable information will not be disclosed in the presentation, report, or publication and will not be stored after completion of the study. The information that you give us in this study will be kept confidential within the study research team. For ensuring your privacy, all discussions that we will have with you will be held in a safe place where no one can hear what we are discussing. Your daily SMS text messages and weekly phone calls, to be delivered for 2 months, will not be accessed by anybody other than the study research team and our record of your phone number will be deleted after the study is completed. In your medication refill visits to the clinic, a research team member will ask you whether you are receiving the intervention regularly if you are assigned into the intervention group.

There are no additional costs to you associated with participating in this research project, nor will you be paid. All SMS and phone calls within the intervention and tests required as part of the research project will be provided to you free of charge.

4 What do I have to do?

All participants in the study will follow their regular anti-Tuberculosis treatment for six months period as per standard care, in whichever study group they assigned. Once you have voluntarily agreed to be involved in the study and been assigned into the intervention group, you will accept to receive the daily SMS text and weekly phone calls medications intake and refill visits reminders for two months. If you are assigned to the no-intervention (control) group, you will still be monitored for those two months.

You will need to disclose your own mobile phone number to the research team to receive an SMS text or phone calls. If you don’t have your own mobile phone, you will need to be able to use your trusted family member’s mobile phone number in the household with a collaborative agreement, to receive the intervention. Participants are expected to read and answer the daily and weekly medications intake and refill visits reminders of SMS text message and phone call, and regularly fellow the information as directed by mobile SMS text or phone calls. Participants need to report to the research team if they change their mobile phone or lose their mobile phone, to immediately replace the old mobile phone number by the new one. You must not forward, tell or show your mobile SMS text and phone call information to other participants as this will affect the study.

5 Other relevant information about the research project

A total of 36 government health centres from Addis Ababa, Ethiopia will be included in the study and a total of 186 participants will be expected to be involved in the study. Of 186 participants, 93 participants will be assigned in the intervention group (SMS text plus phone call reminders). This intervention has been systematically developed through the involvement of different participants such as Tuberculosis infected person, health care providers, health care managers, and Tuberculosis experts. It has also been evaluated for the acceptability and feasibility of the intervention.

6 Do I have to take part in this research project?

Participation in this research project is voluntary. If you do not wish to take part in this study, you do not have to. If you decide to take part in this study and later change your mind, you are free to withdraw from the project at any stage. If you do decide to take part in this study, you will be asked to sign this Participant Information and Consent Form and you will be given a copy to keep with you. Your decision whether to take part in this study or not to take part in this study, to take part and then withdraw, will not affect your routine standard treatment care, your relationship with healthcare professionals treating you or your relationship with this health facility.

7 What are the alternatives to participation?

This is an additional behavioural intervention delivered through mobile SMS text and phone call that evaluates the effectiveness of medication adherence improvement. It is not an alternative treatment to standard care. Your study healthcare provider will discuss this aspect with you before you decide whether or not to take part in this research project.

8 What are the possible benefits of taking part?

We cannot guarantee or promise that you will receive any benefits from this research; however, possible benefits may include participants receiving additional information through daily SMS text messages and weekly phone calls, to remind you to take your daily medications and to attend your medications refill visits. The study may provide relevant evidence for program managers and policymakers to improve Tuberculosis treatment adherence in the future.

9 What are the possible risks and disadvantages of taking part?

This research has no anticipated risk for you, or your family, or your Tuberculosis treatment services you get in future from this health facility. The research has no intention to cause any physical harm, social discrimination, psychological trauma and economic loss. You may feel that some of the questions we ask are stressful or upsetting. If you do not wish to answer a question, you may skip it and go to the next question, or you may stop immediately. If you become upset or distressed as a result of your participation in the research, the study doctor will be able to arrange for counselling or other appropriate support. Any counselling or support will be provided by qualified staff who are not members of the research project team. This counselling service will be provided to you free of charge.

10 What will happen to my test samples?

The proposed sample urine test is part of the medication adherence assessment and will be taken at a random point during months 3 to 4 of your treatment. You will receive information how sample of urine collected before a urine test is made. You will also receive medical advice from the research team after a urine test is completed. Your urine sample will be individually re-identifiable when the test procedure is undertaken, but it will not be stored or transferred for further tests. This study will also collect data from your health record about your treatment adherence. Only non-identifiable information or data about you will be stored for the study. Thus, your urine test and treatment adherence results will be confidential, known only by you and the person who has tested your urine. Signing the consent form means that you agree to have this testing; it will not be done without your consent.

11 What if new information arises during this research project?

Sometimes during the course of a research project, new information becomes available about the intervention that is being studied. If this happens, your study doctor or health care provider will tell you about it and discuss with you whether you want to continue in the research project. If you

decide to continue in the research project you will be asked to sign an updated consent form. On receiving new information, your study doctor or health care provider might consider it to be in your best interests to withdraw you from the research project. If this happens, he/she will explain the reasons and arrange for your regular standard of healthcare to continue.

12 Can I have other treatments during this research project?

Whilst you are participating in this research project, you may not be able to a part of any other behavioural interventional research. It is important to tell your study healthcare provider and the study staff about if you are already enrolled or may want to enrol in another research project.

13 What if I withdraw from this research project?

If you decide to withdraw from this research project, please notify a member of the research team before you withdraw. This notice will allow that the research supervisor to discuss with you about any health risks or special requirements linked to withdrawing If you do withdraw your consent during the research project, the study healthcare provider and relevant study staff will not collect additional personal information from you, although personal information already collected will be retained to ensure that the results of the research project can be measured properly and to comply with the law. You should be aware that data collected by the research team up to the time you withdraw will form part of the research project results. If you do not want them to do this, you must tell the research team when you withdraw from the research project.

14 Could this research project be stopped unexpectedly?

This research project may be stopped unexpectedly for a variety of reasons. These may include reasons such as decisions made by local regulatory/health authorities or by the project sponsor.

15 What happens when the research project ends?

You will finish the study after 2 months of intervention period. You will be asked whether you wish to get a summary of the research work and be informed about how you can access the research results. The research outcomes will be also disseminated to the Federal Ministry of Health, Addis Ababa Health Bureau, and respective government health facilities for decision making for programme improvement and informing future research directions.

Part 2 How is the research project being conducted?

16 What will happen to information about me?

By signing the consent form, you consent to the study research staff collecting and using personal information about you for the research project. Any information obtained in connection with this research project that can identify you will remain confidential. All participant’s information or data related to the research project will keep in the university server and investigators computer and backup electronic file with a protected password and lock cabinet storage for the hard-copy document file. All identifiable information will be removed and replaced by code. All participant information or data will be encrypted. All participant’s identifiable information or data will not be reported or transferred to others. Non-identifiable information will be stored for 5 years after the study in Flinders University repository server and will be destroyed after that period. Your information will only be used for this research project and it will only be disclosed with your permission, except as required by law.

Your health records and any information obtained during the research project are subject to inspection (to verify the procedures and the data) by the relevant authorities and authorised representatives of the project sponsor [Flinders University], by the institution relevant to this

Participant Information Sheet, [South Adelaide clinical HREC], or as required by law. By signing the Consent Form, you authorise the release of, or access to, this confidential information to the relevant study personnel and regulatory authorities as noted above.

It is anticipated that the results of this research project will be published and/or presented in a variety of forums. In any publication and/or presentation, the information will be provided in such a way that you cannot be identified, except with your permission. Your identifiable information or data will not be reported or published. Any information obtained for this research project and for the future research described in Section 16 that can identify you will be treated as confidential and securely stored. It will be disclosed only with your permission, or as required by law.

You have the right to request access to your information collected and stored by the research team. You also have the right to request that any information with which you disagree be corrected. Please contact the study team member named at the end of this document if you would like to access your information.

17 Complaints and compensation

If you suffer any distress or psychological injury as a result of this research project, you should contact the research team as soon as possible. You will be assisted with arranging appropriate counselling support.

18 Who is organising and funding the research?

This research project is being conducted by Zekariyas Sahile, a Doctor of Philosophy candidate of Flinders University. It is being financially supported by Flinders University, Australia and Ambo University, Ethiopia.

No member of the research team will receive a personal financial benefit from your involvement in this research project (other than their ordinary wages). The research investigators declare that have no conflict of interest.

19 Who has reviewed the research project?

All research in Australia involving humans is reviewed by an independent group of people called a Human Research Ethics Committee (HREC). The ethical aspects of this research project have been approved by the South Adelaide Clinical Human Research Ethics Committee and Addis Ababa Health Bureau HREC, Ethiopia.

This project will be carried out according to the Australian National Statement on Ethical Conduct in Human Research (2007). This statement has been developed to protect the interests of people who agree to participate in human research studies.

20 Further information and who to contact

The person you may need to contact will depend on the nature of your query.

If you want any further information concerning this project or if you have any medical problems which may be related to your involvement in the project (for example, any side effects), you can contact the associate investigator on +251911072963 or any of the following people:

Clinical contact person

Name	[Name]
Position	[Position]
Telephone	[Phone number]
Email	[Email address]

For matters relating to research at the site at which you are participating, the details of the local site complaints person are:

Complaints contact person

Name	Zekariyas Sahile
Position	Associate Investigator
Telephone	+251911072963
Email	Neze0002@flinders.edu.au

If you have any complaints about any aspect of the project, the way it is being conducted or any questions about being a research participant in general, then you may contact:

Reviewing HREC approving this research and HREC Executive Officer details

Reviewing HREC name	Addis Ababa Health Burea Human Research Ethics Committee
HREC Executive Officer	Dr Yohannes Channea
Telephone	+2519 11384599
Email	yoha2wok@yahoo.com

Local HREC Office contact (Single Site Research Governance Officer)

Name	Dr Yohannes Channea
Position	HREC-coordinator Addis Ababa Health Bureau, Ethiopia
Telephone	+2519 11384599
Email	yoha2wok@yahoo.com

Consent Form - Adult providing own consent

**Title**	Mobile-assisted Medication Adherence Support (Ma-MAS) intervention for Tuberculosis Patients: Adoption of Intention, Effectiveness, and Experiences in Addis Ababa, Ethiopia.
**Sub-title**	Mobile-assisted medication adherence support (Ma-MAS) Intervention Effectiveness among Tuberculosis patients
**Short Title**	Ma-MAS intervention Effectiveness among Tuberculosis patients
**Protocol Number**	PACTR202002831201865
**Project Sponsor**	Flinders University, Australia
**Coordinating Principal Investigator/ Principal Investigator**	Professor Paul Arbon
**Associate**	Zekariyas Sahile; Dr Lua Perinmal-Lewis Professor Anthony
**Location**	Addis Ababa, Ethiopia

Declaration by Participant

I have read the Participant Information Sheet or someone has read it to me in a language that I understand.

I understand the purposes, procedures and risks of the research described in the project.

I give permission for my doctors, other health professionals, health facility to release information to Flinders University concerning my disease and treatment for the purposes of this research project. I understand that such information will remain confidential.

I have had an opportunity to ask questions and I am satisfied with the answers I have received.

I freely agree to participate in this research project as described and understand that I am free to withdraw at any time during the study without affecting my current or future health care.

I understand that I will be given a signed copy of this document to keep.

Name of Participant (please print) ______________________________

Signature ______________________ Date ______________________

Name of Witness* to
Participant's Signature (please print) ______________________________

Signature ______________________ Date ______________________

* Witness is not to be the investigator, a member of the study team or their delegate. In the event that an interpreter is used, the interpreter may not act as a witness to the consent process. Witness must be 18 years or older.

Declaration by Study Doctor/Senior Researcher†

I have given a verbal explanation of the research project, its procedures and risks and I believe that the participant has understood that explanation.

Name of Study Doctor/
Senior Researcher† (please print) ______________________________

Signature ______________________ Date ______________________

† A senior member of the research team must provide the explanation of, and information concerning, the research project.

Note: All parties signing the consent section must date their own signature.

Form for Withdrawal of Participation - *Adult providing own consent*

It is recommended that this form NOT be included as part of the PICF itself, but that it be developed at the same time and made available to researchers for later use, if necessary. Note that a participant's decision to withdraw their separate consent to the use and storage of tissue will need to be documented separately and linked to the PICF used for that purpose.

**Title**	Mobile-assisted Medication Adherence Support (Ma-MAS) intervention for Tuberculosis Patients: Adoption of Intention, Effectiveness, and Experiences in Addis Ababa, Ethiopia.
**Sub-title**	Mobile-assisted medication adherence support (Ma-MAS) Intervention Effectiveness among Tuberculosis patients
**Short Title**	Ma-MAS intervention Effectiveness among Tuberculosis patients
**Protocol Number**	PACTR202002831201865
**Project Sponsor**	Flinders University, Australia
**Coordinating Principal Investigator/ Principal Investigator**	Professor Paul Arbon
**Associate Investigator(s)**	Zekariyas Sahile; Dr Lua Perimal-Lewis, Professor Anthony Maeder
**Location**	Addis Ababa, Ethiopia

Declaration by Participant

I wish to withdraw from participation in the above research project and understand that such withdrawal will not affect my routine standard care treatment, my relationship with those treating me or my relationship with this health facility.

Name of Participant (please print) ______________________________

Signature ______________________ Date ______________________

In the event that the participant's decision to withdraw is communicated verbally, the Study Doctor/Senior Researcher will need to provide a description of the circumstances below.

Declaration by Study Doctor/Senior Researcher†

I have given a verbal explanation of the implications of withdrawal from the research project and I believe that the participant has understood that explanation.

Name of Study Doctor/
Senior Researcher† (please print) ______________________________

Signature ______________________ Date ______________________

† A senior member of the research team must provide the explanation of and information concerning withdrawal from the research project.

Note: All parties signing the consent section must date their own signature.
