# Supplementary material for: Protocol of a parallel group Randomized Control Trial (RCT) for Mobile-assisted Medication Adherence Support (Ma-MAS) intervention among Tuberculosis patients
Source: PLoS One. 2021 Dec 31;16(12):e0261758. doi: 10.1371/journal.pone.0261758 (PMC8719740; doi:10.1371/journal.pone.0261758)
Supplement: S2 File — (PDF) [file pone.0261758.s006.pdf]

**Project Title:** Mobile-assisted Medication Adherence Support (Ma-MAS) intervention for Tuberculosis Patients: Adoption of Intention, Effectiveness, and Experiences in Addis Ababa, Ethiopia.

Project team roles and responsibilities of investigators and other key project team members.

The person listed as the Chief Investigator / Principal Investigator is responsible for the conduct of the research and listed study staff until completion of the project.

Name: Professor Paul Aron	
Institutional affiliation: Flinders University	
What is the position of this person on the research project? Primary supervisor	
What are the research activities this person will be responsible for: supervise the design of the research proposal, conduct of overall research activity, analysis and interpretation of the findings	
Department and department address: College of Nursing and Health Sciences	
Contact details: a Health or University email address must be used<br>☒ I am the contact person for this project	Phone:+61418856560<br>Email: paul.arbon@flinders.edu.au

Name: Professor Anthony Maeder	
Institutional affiliation: Flinders University	
What is the position of this person on the research project? Primary supervisor	
What are the research activities this person will be responsible for: supervise the design of the research proposal, conduct of overall research activity, analysis and interpretation of the findings	
Department and department address: College of Nursing and Health Sciences	
Contact details: a Health or University email address must be used<br>☐ I am the contact person for this project	Phone: +61882013107<br>Email: anthony.maeder@flinders.edu.au

Name: Dr Lua Perimal-Lewis	
Institutional affiliation: Flinders University	
What is the position of this person on the research project? Co-supervisor	
What are the research activities this person will be responsible for: supervising the design of the research proposal, conduct of overall research activity, analysis and interpretation of the findings	
Department and department address: College of Science and Engineering	
Contact details: a Health or University email address must be used	Phone: +61 8 8201 2069<br>Email: lua.perimal-lewis@flinders.edu.au

☐ I am the contact person for this project	

Name: Zekariyas Sahile Nezenega	
Institutional affiliation: Flinders University	
What is the position of this person on the research project? Associate investigator	
What are the research activities this person will be responsible for: Design the research proposal, conduct overall research activity, analyse and interperate the findings	
Department and department address: College of Nursing and Health sciences	
Contact details: a Health or University email address is preferred	Phone: +61449962325<br>Email: zekariyas.nezenega@flinders.edu.au

Resources

What resources are necessary for the project to be conducted?<br>Human resource, Laptop computer, SMS open software, Isoscreen test kit, audio recorder, smartphone or tablet phone for electronic data collection and stationary.
Please declare what funding support and the amount is being sought or has been secured for this project**:** The research work will be supported by Flinders University and Ambo University Ethiopia. This research project has 9,000 AUD (6,000 AUD for student research support and 3,000 AUD for travel and fieldwork) from Flinders University.

Background

Please refer to the National Statement Chapter 3.1 Elements of Research for guidance on how to ensure this research is conducted in line with core ethical principles.

**Hypothesis** - What is the scientifically valid research question being asked?<br>Hypothesis: Mobile-assisted adherence support intervention improves Tuberculosis medication adherence in addition to routine standard DOT care.
**Aims** - What do the investigators intend to achieve with this research project?<br>To systematically develop, implement and evaluate a mobile-assisted medication adherence support intervention for Tuberculosis patients, and assess its feasibility, intention to adopt, effectiveness, and patient's experiences.
**Objectives** - How will investigators achieve the aims of the research project?<br>Objective-1: To systematically develop mobile-assisted medication adherence support intervention for Tuberculosis patients.<br>Objective-2: To assess the feasibility and acceptability of mobile-assisted medication adherence support intervention.<br>Objective-3: To measure the effect of a mobile-assisted medication adherence support intervention on patients' treatment adherence.<br>Objective-4: To assess patient's perceptions, experiences and intention to adopt a mobile-assisted medication adherence support intervention.
**Expected outcomes** - What do the investigators anticipate the outcomes of this research will be?

This research project will have the following expected outcomes:

- a mobile-assisted medication adherence support intervention for TB patients will be developed using evidence-based and behavioral theories.
- generate evidence on feasibility and acceptability of a mobile-assisted medication adherence support intervention.
- clinical trial results will be used to inform intervention effectiveness on TB medication adherence
- investigate the patient's perception, experiences and intention to adopt a mobile-assisted medication adherence support intervention

Rationale / justification - How the research will fill any gaps and/or contribute to the field of research or contribute to existing or improved practice:

This research study may have the following broader positive effects:

- (R1) the intervention may supplement the effort of the Ministry of Health by designing a mobile-assisted medication adherence support intervention systematically with the local evidence, needs, and practices that may promote medication adherence and treatment outcomes,
- (R2) the study will identify a low-cost validated indirect method adherence measurement tool that can be used along with a non-daily DOT program.
- (R3) if SMS and phone call reminders can to improve treatment adherence and then treatment outcomes, this may have implications in reducing the rates of disease relapse, acquired drug resistance and transmission of infection.

Thus, this project is believed to make a major potential contribution to the tuberculosis treatment plan for the Ethiopian health sector, and globally. The evidence generated from this study will be valuable for policymakers, program managers, health care providers and NGOs working in Ethiopia and beyond.

Literature review Rather than provide a list of references, please explain to the committee how the literature review demonstrates the originality and relevance of your research.

Although 'Bacille Calmette-Guerin' (BCG) vaccine has been used in most parts of the world, there have been doubts of effectiveness (1). Tuberculosis (TB) remains a major global health problem, responsible for ill-health among millions of people each year. According to 2019 WHO's global TB report, in 2018 a TB incidence of 151 per 100,000 population was detected in Ethiopia (2). Non-adherence to TB treatment is a risk factor for further transmission, treatment failure, relapse, acquired multi-drug resistance or extensively drug-resistant tuberculosis and death (3-5). In Ethiopia, non-adherence is a serious threat to TB prevention and control programs. A meta-analysis conducted in Ethiopia found that the pooled prevalence of non-adherence was 21.3% (6).

In Ethiopia, non-adherence to TB treatment is influenced by many factors (7-20). These factors are related to:

- patient-centred factors such as patient's knowledge of TB and its treatment, forgetfulness, perceived risk and wellness, perceived barrier over benefits and psychological distress;
- economic-related factors such as economic constraints, lack adequate food, and employment status;
- social-related factors such as social support, stigma, and discrimination, beliefs in traditional healing;

- health system-related factors such as poor patient-health care provider communication, health information, waiting time and patient satisfaction;
- therapy-related factors such as pills burden, drug side effect, symptom persistence, TB-HIV-coinfection, being at continuation phase of treatment;
- geographical access related factors such as traveling distance to the health facility, traveling cost and time and
- lifestyle-related factors such as the use of alcohol and smoking.

To address these problems of non-adherence to TB medication, World Health Organization (WHO) recommended Direct Observation of Treatment (DOT) by a trained supervisor in which a health worker or a tuberculosis treatment supporter watches the patient take their antibiotics every day to ensure adherence to treatment (21). However, evidence showed that DOT does not provide a solution to poor adherence in TB treatment. For example, in a recent systematic review and meta-analysis found that the magnitude of difference between DOT and Self – Administered Therapy (SAT) on lost to follow-up, treatment failure, cure, treatment completion, and mortality is small and not statistically significant (22-23). Evidence from Ethiopia also indicated that implementing DOT is challenging to patients as it requires patients' daily visits to the health facility which has undesired implications in their work, social life, and high transportation cost. Health professionals also claimed the difficulties of implementing a daily basis DOT at the health facility level. Thus, in Ethiopia DOT has not been fully implemented throughout the treatment regimen (24-25).

Interventions to promote adherence need to touch multiple components to target the barriers to adherence. Interventions that improve patients medication adherence can be categorized into one of four interventions: (1) patient education, (2) improved dosing schedules such as the use of pillbox to organize daily dose, simplifying treatment regimen and reminders to take medications, (3) health service intervention such as increased hours when clinics are open and providing the service in shorter waiting times, and (4) improve health care worker-patients communication (26). In this regard, a mobile health intervention has multiple benefits that facilitate awareness creation through using SMS text and voice calls, remind the patient to take their medication, facilitate easy communication between health care providers and patient's relationship, and enable the service to reach the patients where they live. Thus, It is recommended to use digital health communication like SMS and phone calls to improve TB medication adherence. Mobile phones are increasingly accessible even to those living on US$1 per day in high burden countries (5). In Ethiopia, access to mobile phones is expanding widely: according to the Ethiopia Demography Health Survey (EDHS) 2016, 88% of urban households and 47% of rural households had mobile phone access (27) and this number has since grown when compare to the EDHS 2011, 65% of urban households and 13% rural households have mobile phone

Mobile technology using SMS text interventions has been used for reminders, awareness creation, and behavior change purposes. However, there is contradictory evidence of SMS text intervention effects on treatment adherence and outcomes. Some studies showed that SMS reminders did not provide an improvement on TB medication adherence and treatment outcomes (28-33), while other studies found SMS reminders were effective in clinic attendance and treatment completion, increasing TB cure rate and smear conversion rate, improving treatment completion and a lower rate of interrupted treatment and missed doses, and reducing patients being lost to follow-up (34-36). It is also suggested that the use of a phone call to the patient along with SMS for medication reminders may enhance the relationship between health care provider and patient and treatment compliance. The WHO proposed investigating creatively how SMS can influence treatment adherence by combining with other

digital solutions such as through using voice calls or video technology (37).

Medical Research Council (MRC) guidelines recommend using appropriate theory and evidence when developing an intervention (38-39). However, most adherence interventions are developed without a theoretical base which may be one of the reasons they have not been effective. Literature indicates that the ineffectiveness of specifically mobile health SMS based interventions might be due to the intervention not being developed systematically, not specified and reported, and not designed to the local context and population needs (40-41). Thus, mobile-assisted adherence interventions need to be developed systematically and assessed for effectiveness through rigorous research, to bring quality evidence.

The scientific base for designing SMS and phone call based adherence interventions is founded on the premise that adherence information and motivation can make positive effects on adherence, as it is mediated through behavioural skills that have direct positive effects on adherence and for health education and awareness (42 43).

In Ethiopia one previously conducted randomized trial found that face to face psychological counselling and educational intervention significantly improved patient's treatment adherence. There is also another trial that has been undertaken in Ethiopia that investigates the effect of daily SMS and weekly pill refill reminders on a patient's medication adherence, but the combination of SMS and phone call interventions has not been previously designed and tested. This intervention will be developed systematically, based on the well known MRC framework and behavioral theories. Furtherly, this study will apply the combination of direct and indirect methods of adherence measurements for outcomes assessment that has not been applied in Ethiopia previously.

Project design

Research project setting - i.e. Where is the research being conducted i.e. FMC, GP Plus Marion, Noarlunga Hospital, Tonsley building, Sturt Campus, online forums and alternatives

Please ensure you list the site plus clinic/department/service

The study will be conducted at the primary public health facilities of Addis Ababa, Ethiopia.

Methodological approach - Rationale for choices of methods that are tied back to the aims/objectives:

The Mobile-assisted adherence support intervention will be developed using the MRC framework with embedded mixed-methods, through review of literature and conduct of qualitative research with a phenomenology approach. An in-depth interview technique will be conducted among different stakeholder groups such as Tuberculosis patients, health care professionals, and health care managers.

To generate quality evidence from the trial of a mobile-assisted medication adherence support (Ma-MAS) intervention, the intervention itself needs to be developed systematically with the best available evidence in the context area and appropriate behavioural theory. Thus, the intervention will be developed and evaluated based on the Medical Research Council (MRC) framework for complex interventions in health care. The adapted development phase of the MRC framework guideline has seven different phases of a nonlinear iterative process:

1. phase 1: problem definition (identification and analysis): it has been defined and identified through literature review and will be supported by qualitative research
2. phase 2: systematically identifying the evidence: it has been summarized through literature review, qualitative and quantitative research
3. phase 3: identifying and developing a theory: it has been selected by literature review
4. phase 4: determining the needs (recipients and/or providers): it will be assessed by

qualitative research and review of literature
5.phase 5: examining the practice: it will be assessed by qualitative research
6.phase 6: modeling practice and outcome: a sample prototype is developed and will be reviewed by experts
7.phase 7: intervention design: the final version of messages and communication strategy will be produced.

Table 1: Mobile-assisted medication adherence support intervention development process using adapted development phase of MRC framework and the methods.

Development Phase	Problem identification and definition	Identifying the evidence base	Identifying and/or developing theory	Determine the need	Examine practice	Modeling process and outcome	Intervention design
Methods	Review of literature<br>A qualitative research	Review of literature<br>A qualitative research<br>Quantitative research	Review of literature	Review of literature<br>Qualitative research	Qualitative with phenomenology approach	Modeling a prototype of SMS<br>Identify interrelation<br>Experts opinion through Delphi method	Experts opinion<br>Refine the intervention

Through literature review 70 prototype SMS text messages have been developed for expert review and will be refined based on expert comments through Delphi method (Objective 1). The maximum length of SMS will be 160 characters. The weekly phone call reminders will remind patients to take their medication. During a phone call reminder, patients will be communicated information such as “Good morning, did you take your daily medication?” “Do you have a question regarding your medication” and “Good morning, this is to remind you of your daily medication intake”. The SMS text messages and Phone calls will be delivered in the national official language (Amharic). They will be translated into Amharic language by experts independently of the research team and back-translated to English to review and ensure the consistency of the translation. Appropriate corrections will be made. The prototypes of SMS text messages and Phone call transcripts are attached to this application.

A quantitative cross-sectional design will be used to assess the feasibility and acceptability of a mobile-assisted medication adherence support intervention (Objective 2).

A parallel design individual randomized control trial (RCT) with two groups will be employed to investigate the effect of mobile-assisted adherence intervention on a TB patient’s medication adherence as the primary outcome (Objective 3). Patients will be randomly assigned to one of two groups; Arm-1: patients will get a daily medication intake SMS text and weekly phone call reminders; and Arm-2: patients will not get additional intervention but will receive the routine standard care. All patients in the two groups will have an equal 2 months follow-up period.

All TB patients who will be assigned to any group will receive the same standard treatment based on the National Tuberculosis Treatment guideline. Patients will visit a health facility once per week for medication refill during the continuation phase of treatment. An software system will be used to store, monitor and send automated SMS text messages. Global system for mobile (GSM) sim card and short code SMS will be purchased to manage participants and used via Ethiopian Telecommunication. SMS text messages will be monitored and managed in one computer database from one health centre by a trained IT personnel who will be allocated by FMOH at no cost to the project. A weekly phone call reminder will be made from each health facility by a delegated member of the research support team allocated by FMOH

at no cost to the project.

A survey interview and qualitative study with a phenomenological approach will be conducted to assess the perception, experiences and intention to adopt a mobile-assisted medication adherence support intervention using in-depth interviews (objective 4).

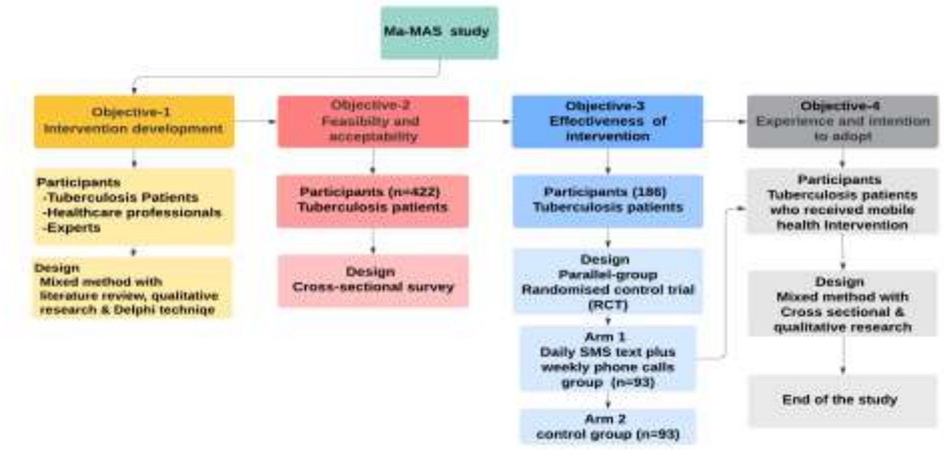

Figure 1: The flow of study objectives

What are your outcome measures?

The patients' medication adherence measured by direct and indirect methods of measurement will be the primary outcome measure of the study. The direct method of adherence measurement using IsoScreen test twice during the intervention (at he first and second months of the intervention) will be performed per the directions provided in the manual. The test result is negative when no colour change is observed after 5 minutes, positive when the colour changed to dark purple and equivocal when the colour turned blue or green. Validated indirect methods of self-report medication adherence will be used along with direct methods using AIDS Clinical Trial Group adherence questionnaire (ACTG) and Visual Analogue Scales (VAS). The classification of adherence to this self-report of adherence will be made based on the direction of measurement tool recommendations. Clinical attendance registration for medication refills visits will also be used for adherence measurement. Patients who delayed at least once for medication refill visits will be considered as non-adherence. The measurement tools will be translated to Amharic by a professional translator and retranslated to English by different expertise independently of the research team to check the consistency of translation and make an appropriate correction if it is found necessary.

Feasibility and acceptability and patient's perceptions, experiences and intention to adopt a mobile-assisted medication adherence support intervention will be the secondary outcome measures of the study.

Project duration: 3 years

Participant selection and activities

How many participants will be selected for the study?

To systematically develop the intervention, the number of in-depth interviews with stakeholder groups will be determined based on the concept of saturation and until no new information is obtained by additional interviews. However, the minimum number of in-depth interviews will not be less than 15.

A total of 422 patients at TB clinics within the public health facilities of Addis Ababa within six months of the commencement of the data collection period will be included for assessing the feasibility and acceptability of a mobile-assisted medication adherence support intervention. Fifty percent (50%) of the primary public health facilities will be included in this study.

A total of 186 participants with arm-1=93, and arm-2=93, will be included in the randomized control trial.

The number of In-depth interviews among patients after the conclusion of the trial will be determined based on the concept of data saturation. The minimum number in-depth interviews will not be less than 15.

All (93) participants in the intervention group will be involved to assess the intention to adopt a mobile-assisted medication adherence support intervention.

How will participants be recruited into the study? – How will they be approached? How are they identified as possible participants?

The recruitment will be made based on the assessment of inclusion and exclusion criteria by a trained research support team of Addis Ababa Health Bureau health professionals. Health professionals at each of the participating clinics will recruit TB participants from their current cohort and health managers in the Addis Ababa Health Bureau will recruit clinical stakeholders.

When developing the intervention we will involve different stakeholder groups:

(1) Tuberculosis patients from primary public health facilities,

(2) primary health care professionals working at TB clinics,

(3) health care system service provider experts and managers working at a sub-city, Addis and Ababa administrative Health Bureau

The above participants will be recruited purposefully for an in-depth interview on ways in which treatment adherence is supported. Information sheets will be sent to these stakeholder groups to participate in the study.

Adult Tuberculosis patients who will be on anti-tuberculosis treatment at primary public health facilities of Addis Ababa, Ethiopia during a specified period of data collection will be included for assessing the feasibility and acceptability of mobile-assisted medication adherence support intervention. Participants will be identified from the registration book, and participant information sheets and a consent form will be given to ask their voluntariness of participation.

Patients enrolled in the intensive phase of anti-tuberculosis treatment will be recruited by the research support team of health care professionals from each health facility based on the eligibility criteria. The recruitment will be continued until the required sample size is obtained. All the recruitment process details will be recorded on paper forms and kept securely by the public health facilities until the project is completed.

What are the inclusion and exclusion criteria?

Inclusion:

All Tuberculosis patients aged 18 and above years and patients whose anti-tuberculosis treatment prescribed for six months are eligible for acceptability and feasibility study.

To be included into the randomized control trial (RCT) participants must be enrolled in a primary public health facility for anti-TB treatment and have attained their first two months of the intensive phase of treatment, aged 18 and above years, have their own mobile phone, and able to read and understand SMS text that is written in the national official language (Amharic). Participants who don't have a mobile phone will also get a chance of inclusion if they have a shared partner's mobile phone in the household with a collaborative agreement.

Exclusion:

Participants will be excluded from the randomized trial if they meet the following conditions:

1. Patients whose anti-tuberculosis treatment has been underway for more than six months.

2. Patients who unable to read and speak the national official language (Amharic) of Ethiopia.

3. Patients who enrolled or agreed to enrol in any other interventional study at the same time as this study is conducted.

All Tuberculosis patients who received a daily SMS text and weekly phone calls for medication intake and refill visits reminders are eligible for perception, experience and adoption of mobile assisted medication adherence support intervention.

Participant commitment -What will their participation involve? i.e. study visits, procedures, tests, tissue samples, questionnaires, wearing of any devices,

Patients will accept the following procedures if voluntarily participating in the study: the data collector assesses the participant's adherence status by taking a urine sample, and asks a few questions on adherence, intention to adopt, socio-demographic, health service-related, substance use, and disease-related information. Participants will also be involved in in-depth interviews during intervention development and in post-intervention In-depth interview to assess their perception and experiences of mobile-assisted medication adherence support intervention. The maximum amount of time participants will spend in contact with the research team is not more than 20 minutes for quantitative data collection and not more than 60 minutes for qualitative interviews. The participant will receive a daily medication intake and weekly phone calls reminders for two months. The details of participant commitment to the research is attached in the Participant Information Sheet and consent form.

Participant follow up – how are participants monitored during the study?

Participants will be monitored whether they are receiving the intervention (SMS text plus phone calls) when they come for medication refill visits to a health facility and during phone calls in every two weeks for two months during the intervention period. The message delivery report will be continuously monitored to ensure the participants have received the SMS text as planned. Participants will also be asked whether they are receiving the intervention every week when they come for their medication refill visit. Participants will be informed about the intervention and instructed not to tell and/or share the SMS text or phone calls with others. For participants who do not answer voice phone calls, three repetitive attempts will be made in a five-minute interval. Participants will be informed clearly to report immediately on any changes in the phone number or lost mobile phone and will be replaced their preferred new phone number subscription. Patients will also be asked if they have a new mobile phone number or if they have changed or lost their phone, during every week of medication refill visits. This is

essential to avoid messaging to the different recipients and for retention, privacy, and confidentiality of the participants.

Consent

**How you will be obtaining consent and/or what alternatives you will be using**:<br>The participants will be informed about the research objectives, procedures, any anticipated risks and benefits of the research in the local official language (Amharic) and 24 hours will be given for them to make a decision to participate in the study. A written informed consent form will be given to participants for their signature for voluntary participation. Participants will be requested to sign a separate written informed consent for each research objective. Participants will be disclosed all procedures applied in the study and how their information will be kept confidential. Participants have the right to withdraw from the study at any time when they feel uncomfortable or unable to continue in the study, and they will be informed of its limitations or consequences on the research during consent requests. The participant will be disclosed data management and storage, any relevant intellectual property and copyright arrangements. The participants will be informed about the result of recent medication adherence at each time of measurement and medical advice will be given on the consequence of non-adherence to medication for all groups of participants. The participants will also be disclosed how the result of the research will be reported and asked whether they wish to receive a summary of the outcome. For detailed information, see the attached participant consent forms.
**Which investigators will issue the information sheets and consent forms**:<br>The principal investigator is responsible to issue the information sheets and consent forms. Participants will be recruited and informed about the research aim with plain language, appropriate culture, and respect by the research support team.
**How much time will participants have to consider participation**:<br>The participants will be given 24 hours to decide on voluntary participation in the study.
**Please specify which investigators will obtain consent from participants**:<br>The principal investigator is responsible to ensure participants have provided consent. A research support team member will obtain consent from each participant.
**Will there be an opportunity to confirm or renegotiate consent during the research project**? – I.e. children or young people are involved, the capacity of participants changes, the terms of consent change, action research methods are used:<br>A separate written consent will be used for each research objective. This research will not involve children or young people or incapacitated participants.
**Who will be confirming or renegotiating consent with participants and what process will be undertaken?**<br>The research team will be confirming or renegotiating consent with participants for each research objective if they are involved in more than one. The principal investigator will be ensuring participants are confirming their consent to participation for each research objective.

Data management – as required in addition to that outlined in your HREA

**Who will collect the study data /information?**

Diploma and above degree healthcare professionals will be recruited for both quantitative and qualitative data collection and supervision based on their previous experience. The recruitment will be conducted through Addis Ababa Health Bureau and they will be responsible for any time allocation and remuneration impacts on their staff, with no cost to the project. A separate group of data collectors and supervisors will be used for quantitative and qualitative studies. A two-day training will be given by the principal investigator for data collectors and supervisors on data collection procedures and quality assurance based on the protocol.

To assess adherence status using the IsoScreen test a research support team member with a laboratory technology background will collect a sample of urine and test the presence of Isoniazid metabolites in urine. The Ministry of Health laboratory facilities will be used for this adherence assessment.

What format will the data or information be stored?

The quantitative data will be collected electronically using ODK in Microsoft excel format.

A sample of urine will not be stored or transferred to third parties for further analysis or studies.

Nonidentifiable data will be stored in Microsoft excel format.

The qualitative data will be kept in audio recorded format and will be transcribed to verbatim.

Please provide details regarding the training of the research team on maintaining the integrity and security of the data

The research team (intervention providers, data collectors and supervisors) will be trained by the principal investigator for two days on the research procedures of the intervention, data collection, the quality and security of the data as per the research protocol.

What conditions can the data be accessed or granted to others?

In the case of a formal request for further research purposes, non-identifiable secondary data may be granted to others. A participant’s identifiable information will be removed and replaced by code to protect the anonymity of the participants. Thus, identifiable data and/or information will not be accessed or granted to others.

How will the research data be stored and what security measures are in place to protect it?

To ensure the security of the patients’ information, all data will keep in the university server and principal investigator laptop computer and backup file. All identifiable information will be removed and replaced by code. The electronic data and research files will be protected by password in the investigator’s computer and Flinders University server. All hard-copy research documents will be locked in cabinet storage at the Addis Ababa Health Bureau facility.

How will you provide access to, disclose, use/re-use or transfer the data?

The secondary data may be available for further research with a formal request to reuse the data. Participant’s identifiable information or data will not be transferred to third party.

How long will the data be retained for? Data should be retained to allow for sufficient time to allow reference to them by other researchers and interested parties.

Data will be retained for 5 years.

What plans are in place to store / archive the study data once the research is completed?

All data will be kept in the University server and investigator's computer and backup file. The electronic data files will be protected using a password and there will be locked storage cabinets in the Addis Ababa Health Bureau facilities for any hard copy records. Participant’s identifiable information will not be stored or transferred to a third party.

**How will the study data be destroyed?**<br>All data will be destroyed after 5 years from the completion of the research project.
**Matching and sampling strategies:**<br>Participants who meet the selection criteria will be randomized into one of the two groups by a computer-generated algorithm. The number of patients allocated from each health facility will be determined based on the number of patients who enrolled in the intensive phase of treatment. A total of 36 primary Public health facilities will be included in the randomized trial with the assumption that on average 6 patients per one public health facility will be obtained.
**Accounting for potential bias, confounding factors and missing information:**<br>Single-blinding will be applied to avoid bias in outcome measurement by differentiating the person who measures the outcome from the person who randomizes and provides the intervention. Thus, the person who measures the outcome is blind to which group participants are assigned. All interviews will be conducted after the patient finishes the non-daily DOT service. Interviews will be conducted in a different room from the TB clinic to be convenient for discussion and privacy. All factors that are potentially confounding factors will be identified through literature review. Confounding factors will be controlled by restriction and multivariable regression analysis. As far as possible to avoid missing information, data will be collected electronically and any further missing information will be identified and analyzed by intention to treat (ITT).
**Sample size and statistical or power issues –** Make sure the size and profile of the sample to be recruited is adequate to answer the research question – please provide details**:**<br>A Stata code *power twoproportions* was used to calculate the sample size considering the primary outcome of TB medication non-adherence. The rate of TB medication non-adherence in Ethiopia varies considerably in the literature range from 10% to 26% depending on the patient's treatment phase and different settings. We found the rate of non-adherence was 25.6% (po=0.256%) during the continuation phase of treatment from the previous study conducted at Addis Ababa, Ethiopia in the control group (12). The absolute reduction of TB non-adherence by 15% due to mobile SMS text intervention found on a similar study (101) yielding a non-adherence rate of 10.6% (pa=0.106) in the intervention group. Considering simple randomised controlled trials parallel-group design, 95% confidence level (alpha=0.05), 80% power (beta=0.80) and a one-sided p-value of 0.025 the initial sample size would yield n=81 participants per arm. Assuming an attrition rate of 15%, the final sample size would become total n=186 with arm-1 n=93, and arm-2 n=93.<br>A total of 422 participants are calculated for feasibility and acceptability study using single population proportion formula considering 95% confidence level (alpha=0.05), Proportion of 50% and 10% non-response rate.
**How will you measure, manipulate and/or analyse the information collected?**<br>An in-depth interview guideline has been developed through review of relevant literature, to systematically develop mobile-assisted medication adherence support (Ma-MAS) intervention and assess the perceptions and experiences of the intervention.<br>To assess the intention to adopt Ma-MAS intervention, a data collection tool that was developed based on unified theory of acceptance and use of technology will be adapted from different literature (44-47). The feasibility of the intervention will be assessed in terms of access to mobile and the capability to use SMS and phone call. The feasibility tool has been adopted from a similar study (48). The most commonly used self-report indirect methods adherence tools, AIDS Clinical Trial Group adherence questionnaire (ACTG) (49)(50) and Visual Analogue Scales (VAS) (51), will be used to measure a patient's medication adherence.

Additionally, medication refill visit attendance will be recorded in every week's visits to a health facility (50). IsoScreen NIH metabolites in urine tests will be used to evaluate medication adherence. All other factors that could be confounding factors have been identified through the literature review and included in the tool.

Tabel 2: Data collection tools and time of measure

Variable	Data collection tool	Time of measure
**Intervention** development	A literature review and a qualitative interview guide	At the beginning of the study
**Feasibilty and acceptability**	A tool developed using literature review and research question	Before the trial started
**Medication adherence**	AIDS Clinical Trial Group adherence questionnaire (ACTG)	At baseline and end of intervention
	Visual Analogue Scales (VAS)	At baseline and end of intervention
	Refill visits attendance	At baseline and end of intervention
	IsoScreen test	At one and two months of intervention
	Demographic and Other factors	At baseline assessement
**Perception and experience**	A Indepth interview Guide	Post intervention (end of intervention)
**Adoption of intention**	Unified theory of acceptance and use of technology (UTAUT)	Post intervention (end of intervention)

The collected data will be cleaned and imported to STATA version 14 for analysis. Mean, SD, and Median will be used for continuous variables, and frequency and percentage for categorical variables. Intention to treat (ITT) analysis will be performed and multivariable regression model analysis will be used to detect the statistical effect of the mobile-assisted adherence intervention. Factor analysis and structural equation modelling (SEM) will be employed to assess the correlation between behavioural intention with constructs of attitude, performance expectancy, effort expectancy, social influence and facilitating conditions factors that affect the adoption of mobile-assisted medication adherence intervention. The indirect method of adherence measure will be validated locally through analyzing sensitivity, specificity, positive predictive value (PPV) and negative predictive value (NPV) and compared with Isoscreen urine test technology. All qualitative data will be transcribed, and the transcript will be analyzed thematically through the application of Nvivo software.

Data linkage – what linkages are planned or anticipated?

Data linkage does not apply to this research project.

What impact will a participant withdrawing have on the data and how will this be responded to?

Participant's withdrawal from the research will have an impact on the trial as it may lead to not have the power to detect the intervention effect. Thus, we have added 15% more on the sample size calculation for loss to follow up participants.

Results, reporting, outcomes and future plans

Please detail your plans for the return of the research results to the participants:
The participants will be asked whether they wish to get a summary of the research work, and if so in what form, and will be informed about how they can access the research results.

What are your plans for dissemination and publication of project outcomes:

The principal investigator is responsible for disseminating the finding to Health facilities, Addis Ababa Health Bureau, Federal Ministry of Health (FMOH), Flinders University, national and international conferences. An effort will be made to publish the research findings in an international journal to reach the scientific community.
**Please detail other potential uses of the data at the end of the project**:<br>The data will be used to answer the research purposes. Besides, the unidentifiable data will be open access in the university repository server to make the data available for re-use for future research purposes.
**What are your plans for sharing and/or future use of data and/or follow-up research?**<br>i.e. anticipated secondary use of data:<br>The data will be available for the use of secondary data in the University repository server for future research. Any identifiable participant's information will not be stored and transferred to the third party.
**What is the project closure process?** :<br>All participants will be acknowledged their voluntary participation in the study by the research team at the end of the study. This study has no forms of inducement, coercion and the study does not bring any risks that incur compensation.

References

1. Ian M Orme, Beyond BCG: the potential for a more effective TB vaccine.Molecular Medicine Today 1999 5(11) page 487- 492
2. World Health Organization. Global tuberculosis report 2019. Geneva: World Health Organization; 2019.
3. Yew WW. Directly observed therapy, short-course: the best way to prevent multidrug-resistant tuberculosis. Chemotherapy. 1999;45(Suppl. 2):26-33.
4. Pablos-Méndez A, Knirsch CA, R Graham Barr M, Lerner BH, Frieden TR. Nonadherence in tuberculosis treatment: predictors and consequences in New York City. The American journal of medicine. 1997;102(2):164-70.
5. Barclay E. Text messages could hasten tuberculosis drug compliance. The Lancet. 2009;373(9657):15-6.
6. Zegeye A, Dessie G, Wagnew F, Gebrie A, Islam SMS, Tesfaye B, et al. Prevalence and determinants of anti-tuberculosis treatment non-adherence in Ethiopia: A systematic review and meta-analysis. PloS one. 2019;14(1):e0210422.
7. Ogundele OA, Moodley D, Seebregts CJ, Pillay AW, editors. An ontology for tuberculosis treatment adherence behaviour. Proceedings of the 2015 Annual Research Conference on South African Institute of Computer Scientists and Information Technologists; 2015: ACM.
8. Woimo TT, Yimer WK, Bati T, Gesesew HA. The prevalence and factors associated for anti-tuberculosis treatment non-adherence among pulmonary tuberculosis patients in public health care facilities in South Ethiopia: a cross-sectional study. BMC public health. 2017;17(1):269.
9. Sahile Z, Yared A, Kaba M. Patients' experiences and perceptions on associates of TB treatment adherence: a qualitative study on DOTS service in public health centers in Addis Ababa, Ethiopia. BMC Public Health. 2018;18(1):462.
10. Adane AA, Alene KA, Koye DN, Zeleke BM. Non-adherence to anti-tuberculosis treatment and determinant factors among patients with tuberculosis in northwest Ethiopia. PloS one. 2013;8(11):e78791.

11. Tesfahuneygn G, Medhin G, Legesse M. Adherence to Anti-tuberculosis treatment and treatment outcomes among tuberculosis patients in Alamata District, northeast Ethiopia. BMC research notes. 2015;8:503.
12. Tola HH, Garmaroudi G, Shojaeizadeh D, Tol A, Yekaninejad MS, Ejeta LT, et al. The effect of psychosocial factors and patients' perception of tuberculosis treatment non-adherence in Addis Ababa, Ethiopia. 2017;27(5):447-8.
13. Gebremariam MK, Bjune GA, Frich JC. Barriers and facilitators of adherence to TB treatment in patients on concomitant TB and HIV treatment: a qualitative study. BMC public health. 2010;10:651.
14. Gugssa Boru C, Shimels T, Bilal AI. Factors contributing to non-adherence with treatment among TB patients in Sodo Woreda, Gurage Zone, Southern Ethiopia: A qualitative study. Journal of infection and public health. 2017;10(5):527-33.
15. Nezenega ZS, Gacho YH, Tafere TE. Patient satisfaction on tuberculosis treatment service and adherence to treatment in public health facilities of Sidama zone, South Ethiopia. BMC health services research. 2013;13:110.
16. Sagbakken M, Frich JC, Bjune G. Barriers and enablers in the management of tuberculosis treatment in Addis Ababa, Ethiopia: a qualitative study. BMC Public Health. 2008;8:11.
17. Getahun B, Nkosi ZZ. Satisfaction of patients with directly observed treatment strategy in Addis Ababa, Ethiopia: A mixed-methods study. PloS one. 2017;12(2):e0171209.
18. Shargie EB, Lindtjørn B. Determinants of treatment adherence among smear-positive pulmonary tuberculosis patients in Southern Ethiopia. PLoS medicine. 2007;4(2):e37.
19. Mekonnen HS, Azagew AW. Non-adherence to anti-tuberculosis treatment, reasons and associated factors among TB patients attending at Gondar town health centers, Northwest Ethiopia. BMC research notes. 2018;11(1):691.
20. Habteyes Hailu T, Azar T, Davoud SHOJAEIZADEH GGJIjoph. Tuberculosis treatment non-adherence and lost to follow up among TB patients with or without HIV in developing countries: a systematic review. 2015;44(1):1.
21. World Health Organization. An expanded DOTS framework for effective tuberculosis control. Int J Tuberc Lung Dis. 2002;6:378-88.
22. Karumbi J, Garner P. Directly observed therapy for treating tuberculosis. Cochrane Database of Systematic Reviews. 2015(5).
23. McKay B, Castellanos M, Ebell M, Whalen CC, Handel A. An attempt to reproduce a previous meta-analysis and a new analysis regarding the impact of directly observed therapy on tuberculosis treatment outcomes. PloS one. 2019;14(5):e0217219.
24. Sagbakken M, Frich JC, Bjune G. Barriers and enablers in the management of tuberculosis treatment in Addis Ababa, Ethiopia: a qualitative study. BMC public health. 2008;8(1):11.
25. Fiseha D, Demissie M. Assessment of Directly Observed Therapy (DOT) following tuberculosis regimen change in Addis Ababa, Ethiopia: a qualitative study. BMC infectious diseases. 2015;15(1):405.
26. Osterberg L, Blaschke TJNEjom. Adherence to medication. 2005;353(5):487-97.
27. Central Statistical Agency CSAE, Icf. Ethiopia Demographic and Health Sruvey 2016. Addis Ababa, Ethiopia: CSA and ICF; 2017.
28. Mohammed S, Glennerster R, Khan AJ. Impact of a daily SMS medication reminder system on tuberculosis treatment outcomes: a randomized controlled trial. PloS one. 2016;11(11):e0162944.

29. Liu X, Lewis JJ, Zhang H, Lu W, Zhang S, Zheng G, et al. Effectiveness of electronic reminders to improve medication adherence in tuberculosis patients: a cluster-randomised trial. PLoS medicine. 2015;12(9):e1001876.
30. Nglazi MD, Bekker L-G, Wood R, Hussey GD, Wiysonge CS. Mobile phone text messaging for promoting adherence to anti-tuberculosis treatment: a systematic review. BMC infectious diseases. 2013;13(1):566.
31. Iribarren S, Beck S, Pearce PF, Chirico C, Etchevarria M, Cardinale D, et al. TextTB: a mixed method pilot study evaluating acceptance, feasibility, and exploring initial efficacy of a text messaging intervention to support TB treatment adherence. 2013;2013.
32. Bediang G, Stoll B, Elia N, Abena JL, Geissbuhler A. SMS reminders to improve adherence and cure of tuberculosis patients in Cameroon (TB-SMS Cameroon): a randomised controlled trial. BMC Public Health. 2018;18(1):583.
33. Liu X, Lewis JJ, Zhang H, Lu W, Zhang S, Zheng G, et al. Effectiveness of Electronic Reminders to Improve Medication Adherence in Tuberculosis Patients: A Cluster-Randomised Trial. PLoS Med. 2015;12(9):e1001876.
34. Nglazi MD, Bekker L-G, Wood R, Hussey GD, Wiysonge CS. Mobile phone text messaging for promoting adherence to anti-tuberculosis treatment: a systematic review. BMC Infectious Diseases 2013;13:566.
35. Fang XH, Guan SY, Tang L, Tao FB, Zou Z, Wang JX, et al. Effect of short message service on management of pulmonary tuberculosis patients in Anhui Province, China: A prospective, randomized, controlled study. Medical Science Monitor. 2017;23:2465-9.
36. Broomhead S, Mars MJT, e-Health. Retrospective return on investment analysis of an electronic treatment adherence device piloted in the Northern Cape Province. 2012;18(1):24-31.
37. World Health Organization. Handbook for the use of digital technologies to support tuberculosis medication adherence. World Health Organization; 2017. Report No.: 9241513454.
38. Craig P, Dieppe P, Macintyre S, Michie S, Nazareth I, Petticrew MJB. Developing and evaluating complex interventions: the new Medical Research Council guidance. 2008;337:a1655.
39. Lakshman R, Griffin S, Hardeman W, Schiff A, Kinmonth AL, Ong KK. Using the Medical Research Council framework for the development and evaluation of complex interventions in a theory-based infant feeding intervention to prevent childhood obesity: the baby milk intervention and trial. Journal of obesity. 2014;2014:646504.
40. Horne R, Weinman J, Barber N, Elliott R, Morgan M, Cribb A, et al. Concordance, adherence and compliance in medicine taking. 2005;2005:40-6.
41. Campbell NC, Murray E, Darbyshire J, Emery J, Farmer A, Griffiths F, et al. Designing and evaluating complex interventions to improve health care. BMJ. 2007;334(75991):455-9.
42. Alegria-Flores K, Weiner BJ, Wiesen CA, Lich KLH, Van Rie A, Paul JE, et al. Innovative approach to the design and evaluation of treatment adherence interventions for drug-resistant TB. The international journal of tuberculosis and lung disease : the official journal of the International Union against Tuberculosis and Lung Disease. 2017;21(11):1160-6.
43. Aranda-Jan CB, Mohutsiwa-Dibe N, Loukanova SJBph. Systematic review on what works, what does not work and why of implementation of mobile health (mHealth) projects in Africa. 2014;14(1):188.
44. Zhang X, Zaman Bu. Adoption mechanism of telemedicine in underdeveloped country. Health Informatics Journal. 2019:1460458219868353.

45. Davis FDJMq. Perceived usefulness, perceived ease of use, and user acceptance of information technology. 1989:319-40.
46. Dwivedi YK, Shareef MA, Simintiras AC, Lal B, Weerakkody VJGIQ. A generalised adoption model for services: A cross-country comparison of mobile health (m-health). 2016;33(1):174-87.
47. Venkatesh V, Morris MG, Davis GB, Davis FDJMq. User acceptance of information technology: Toward a unified view. 2003:425-78.
48. Bobrow K, Farmer A, Cishe N, Nwagi N, Namane M, Brennan TP, et al. Using the Medical Research Council framework for development and evaluation of complex interventions in a low resource setting to develop a theory-based treatment support intervention delivered via SMS text message to improve blood pressure control. BMC health services research. 2018;18(1):33.
49. Chesney MA, Ickovics J, Chambers D, Gifford A, Neidig J, Zwickl B, et al. Self-reported adherence to antiretroviral medications among participants in HIV clinical trials: the AACTG adherence instruments. 2000;12(3):255-66.
50. Boogaard Jvd, Lyimo RA, Boeree MJ, Kibiki GS, Aarnoutse RE. Electronic monitoring of treatment adherence and validation of alternative adherence measures in tuberculosis patients: a pilot study. Bulletin of the World Health Organization. 2011;89:632-9.
51. Kalichman SC, Amaral CM, Swetzes C, Jones M, Macy R, Kalichman MO, et al. A simple single-item rating scale to measure medication adherence: further evidence for convergent validity. 2009;8(6):367-74.
