# Supplementary material for: Protocol of a parallel group Randomized Control Trial (RCT) for Mobile-assisted Medication Adherence Support (Ma-MAS) intervention among Tuberculosis patients
Source: PLoS One. 2021 Dec 31;16(12):e0261758. doi: 10.1371/journal.pone.0261758 (PMC8719740; doi:10.1371/journal.pone.0261758)
Supplement: S3 File — (PDF) [file pone.0261758.s007.pdf]

Pan African Clinical Trials Registry

South African Medical Research Council, South African Cochrane Centre
PO Box 19070, Tygerberg, 7505, South Africa
Telephone: +27 21 938 0506 / +27 21 938 0834 Fax: +27 21 938 0836
Email: pactradmin@mrc.ac.za Website: www.pactr.org

**Trial no.:**	PACTR202002831201865	**Date of Approval:**	18/02/2020
**Trial Status:**	Registered in accordance with WHO and ICMJE standards		

TRIAL DESCRIPTION

**Public title**	Mobile-assisted Medication Adherence Support (Ma-MAS) Intervention for Tuberculosis Patients in Addis Ababa, Ethiopia.
**Official scientific title**	Mobile-assisted Medication Adherence Support (Ma-MAS) Intervention among Tuberculosis Patients in Addis Ababa, Ethiopia; Randomized Control Trial (RCT)
**Brief summary describing the background and objectives of the trial**	Non-adherence to TB treatment is a risk factor for further transmission, treatment failure, relapse, acquired multi-drug resistance or extensively drug-resistant tuberculosis and death. In Ethiopia, non-adherence is a serious threat to TB prevention and control programs. There are various factors affecting non-adherence behaviour. Lack of information and skill for self-management, motivation, and self-efficacy are the major barriers to adherence. The widespread expansion of mobile phones provides opportunities to address challenges related to non-adherence, as mobile health can facilitate direct communication between health care providers and patients through using SMS and voice phone calls. However, the existing evidence is inconsistent about the effect of SMS text on TB treatment adherence and outcomes. Because the evidence on mobile interventions is equivocal, there is a reason to try and establish it more rigorously, by developing the SMS text intervention systematically with evidence-based and conducting the trial with strong measurement methods. Thus, this study aims to investigate the effect of mobile-assisted adherence support intervention using SMS text and Phone calls on TB medication adherence.
**Type of trial**	RCT
**Acronym (If the trial has an acronym then please provide)**	
**Disease(s) or condition(s) being studied**	Infections and Infestations
**Sub-Disease(s) or condition(s) being studied**	Tuberculosis
**Purpose of the trial**	Behavioural Intervention
**Anticipated trial start date**	31/03/2021
**Actual trial start date**	
**Anticipated date of last follow up**	31/07/2021
**Actual Last follow-up date**	
**Anticipated target sample size (number of participants)**	186
**Actual target sample size (number of participants)**	
**Recruitment status**	Not yet recruiting
**Publication URL**	

**Secondary Ids**	**Issuing authority/Trial register**

STUDY DESIGN

**Intervention assignment**	**Allocation to intervention**	**If randomised, describe how the allocation sequence was generated**	**Describe how the allocation sequence/code was concealed from the person allocating the participants to the intervention arms**	**Masking**	**If masking / blinding was used**
Parallel: different groups receive	Randomised	Simple randomization using a randomization table	Central randomisation by phone/fax	Masking/ blinding used	Care giver/Provider,

different interventions at same time during study		created by a computer software program			Outcome Assessors

INTERVENTIONS

Intervention type	Intervention name	Dose	Duration	Intervention description	Group size	Nature of control
Experimental Group	SMS text plus phone calls	A daily SMS text and weekly phone calls for medication intake and medication refill visits reminders	2 months	Arm-1 (intervention) group participants will receive a daily SMS text and weekly phone calls for medication intake and medication refill visit reminders	93	
Control Group	Non daily DOT alone		2 months	Arm-2 (control) group participants will receive the same routine standard treatment care as the intervention group, but not receive the additional intervention. Patients in the control group will be followed for an equal period as the intervention group during the continuation phase of treatment.	93	Active-Treatment of Control Group

ELIGIBILITY CRITERIA

List inclusion criteria	List exclusion criteria	Age Category	Minimum age	Maximum age	Gender
To be included into the randomized control trial (RCT) • participants must be enrolled in a primary public health facility for anti-TB treatment and have attained their first two months of the intensive phase of treatment • participants must be aged 18 years and above • participants must be able to read and understand SMS text that is written in the national official language (Amharic) of Ethiopia • participants must have their own mobile phone, or • participants who do not have a mobile phone can be included if they have a shared mobile phone in the household with a collaborative agreement; (i.e. a voluntary agreement between the patient and family member living in the same household).	Participants will be excluded from the randomized trial if they meet the following conditions: Patients whose anti-tuberculosis treatment prescribed for more than six months of treatment. Patients who unable to read and speak the national official language of Ethiopia. Patients who enrolled or agreed to enroll in another trial study as the same time with this study.	80 and over: 80+ Year,Adult: 19 Year-44 Year,Aged: 65+ Year(s),Middle Aged: 45 Year(s)-64 Year(s)	18 Year(s)	80 Year(s)	Both

ETHICS APPROVAL

Has the study received appropriate ethics committee approval	Date the study will be submitted for approval	Date of approval	Name of the ethics committee
Yes		08/04/2020	Southern Adelaide Clinical Human Research Ethics Committee

Ethics Committee Address

Street address	City	Postal code	Country
Flinders Medical centre, Bedford Park, South Australia	Adelaide	5042	Australia

OUTCOMES

Type of outcome	Outcome	Timepoint(s) at which outcome measured

Primary Outcome	TB medication adherence	At the baseline, and at the random point of 4 and 8 weeks of intervention
Secondary Outcome	Nil	Nil

RECRUITMENT CENTRES

Name of recruitment centre	Street address	City	Postal code	Country
Primary health facilities	Addis Ababa	Addis Ababa		Ethiopia

FUNDING SOURCES

Name of source	Street address	City	Postal code	Country
Flinders University	Sturt Rd, Bedford Park SA 5042	Adelaide		Australia
Ambo University	Ambo 01	Ambo		Ethiopia

SPONSORS

Sponsor level	Name	Street address	City	Postal code	Country	Nature of sponsor
Primary Sponsor	Flinders University	Sturt Rd, Bedford Park SA 5042	Adelaide		Australia	University

COLLABORATORS

Name	Street address	City	Postal code	Country

CONTACT PEOPLE

Role	Name	Email	Phone	Street address
**Principal Investigator**	Zekariyas Nezenega	neze0002@flinders.edu.au	+61449962325	40 Cheltenham St, Highgate SA
**City**	**Postal code**	**Country**	**Position/Affiliation**	
Adelaide		Australia	PhD Candidate	
**Role**	**Name**	**Email**	**Phone**	**Street address**
**Scientific Enquiries**	Anthony Maeder	anthony.maeder@flinders.edu.au	61882013107	1284 South Rd, Tonsley SA
**City**	**Postal code**	**Country**	**Position/Affiliation**	
Adelaide		Australia	Professor of Digital Health Systems in Flinders University	
**Role**	**Name**	**Email**	**Phone**	**Street address**
**Public Enquiries**	Yohannes Dr	yoha2wok@yahoo.com	251911384599	Addis Ababa Health Bureau
**City**	**Postal code**	**Country**	**Position/Affiliation**	
Addis Ababa		Ethiopia	Addis Ababa Health Bureau Human Research Ethics committee coordinator	
**Role**	**Name**	**Email**	**Phone**	**Street address**
**Scientific Enquiries**	Lua Perimal Lewis	lua.perimal-lewis@flinders.edu.au	+61882012069	1284 South Rd, Tonsley SA
**City**	**Postal code**	**Country**	**Position/Affiliation**	
Adelaide		Australia	Senior Research Fellow in Digital Health in Flinders University	
**Role**	**Name**	**Email**	**Phone**	**Street address**
**Scientific Enquiries**	Paul Arbon	Paul.arbon@flinders.edu.au	+61418856560	Sturt Road Bedford Park
**City**	**Postal code**	**Country**	**Position/Affiliation**	
Adelaide		Australia	Matthew Flinders Distinguished Professor	

REPORTING

Share IPD	Description	Additional Document	Sharing Time Frame	Key Access Criteria

		Types		
Yes	Summary results or link to summary results will be included within the trials registration records.	Study Protocol	Within 12 months of the study completion date	The study protocol and summary of results will be an open access.
**URL**	**Results Available**	**Results Summary**	**Result Posting Date**	**First Journal Publication Date**
	No			
**Result Upload 1:**	**Result Upload 2:**	**Result Upload 3:**	**Result Upload 4:**	**Result Upload 5:**
**Result URL Hyperlinks**	**Link To Protocol**			
Result URL Hyperlinks				

Changes to trial information

Section Name	Field Name	Date	Reason	Old Value	Updated Value
Trial Information	Trial description	12/02/2020	editorial	Non-adherence to TB treatment is a risk factor for further transmission, treatment failure, relapse, acquired multi-drug resistance or extensively drug-resistant tuberculosis and death. In Ethiopia, non-adherence is a serious threat to TB prevention and control programs. There are various factors affecting non-adherence behaviour. Lack of information and skill for self-management, motivation, and self-efficacy are the major barriers to adherence. The widespread expansion of mobile phones provides opportunities to address challenges related to non-adherence, as mobile health can facilitate direct communication between health care providers and patients through using SMS and voice phone calls. However, the existed evidence is inconsistent about the effect of SMS text on TB treatment adherence and outcomes. Because the evidence on mobile interventions is equivocal, there is reason to try and establish it more rigorously, by developing the SMS text intervention systematically with evidence-based and conducting the trial with strong measurement methods. Thus, this study aims to investigate the effect of mobile-assisted adherence support intervention using SMS text and Phone calls on TB medication adherence.	Non-adherence to TB treatment is a risk factor for further transmission, treatment failure, relapse, acquired multi-drug resistance or extensively drug-resistant tuberculosis and death. In Ethiopia, non-adherence is a serious threat to TB prevention and control programs. There are various factors affecting non-adherence behaviour. Lack of information and skill for self-management, motivation, and self-efficacy are the major barriers to adherence. The widespread expansion of mobile phones provides opportunities to address challenges related to non-adherence, as mobile health can facilitate direct communication between health care providers and patients through using SMS and voice phone calls. However, the existing evidence is inconsistent about the effect of SMS text on TB treatment adherence and outcomes. Because the evidence on mobile interventions is equivocal, there is a reason to try and establish it more rigorously, by developing the SMS text intervention systematically with evidence-based and conducting the trial with strong measurement methods. Thus, this study aims to investigate the effect of mobile-assisted adherence support intervention using SMS text and Phone calls on TB medication adherence.

Section Name	Field Name	Date	Reason	Old Value	Updated Value
Eligibility	Age group	12/02/2020	Age 18 and above year will be included in the study	Adult: 19 Year-44 Year, Middle Aged: 45 Year(s)-64 Year(s), Aged: 65+ Year(s)	Adult: 19 Year-44 Year, Middle Aged: 45 Year(s)-64 Year(s), Aged: 65+ Year(s), 80 and over: 80+ Year
**Section Name**	**Field Name**	**Date**	**Reason**	**Old Value**	**Updated Value**
Eligibility	Maximum age	12/02/2020	Age 18 and above years included in the study	65 Year(s)	80 Year(s)
**Section Name**	**Field Name**	**Date**	**Reason**	**Old Value**	**Updated Value**
Reporting	Plan to share IPD	13/02/2020	IPD sharing statement is needed	No	Yes
**Section Name**	**Field Name**	**Date**	**Reason**	**Old Value**	**Updated Value**
Reporting	IPD description	13/02/2020	IPD sharing statement is needed		Summary results or link to summary results will be included within the trials registration records.
**Section Name**	**Field Name**	**Date**	**Reason**	**Old Value**	**Updated Value**
Reporting	IPD-Sharing time frame	13/02/2020	IPD sharing statement is needed		Within 12 months of the study completion date
**Section Name**	**Field Name**	**Date**	**Reason**	**Old Value**	**Updated Value**
Reporting	Key access criteria	13/02/2020	IPD sharing statement is needed		The study protocol and summary of results will be an open access.
**Section Name**	**Field Name**	**Date**	**Reason**	**Old Value**	**Updated Value**
Reporting	Study protocol document	13/02/2020	IPD sharing statement is needed		Study Protocol
**Section Name**	**Field Name**	**Date**	**Reason**	**Old Value**	**Updated Value**
Recruitment Centre	RecruitmentCentre List	18/02/2020	Editorial	Addis Ababa Primary health facilities , Addis Ababa , Addis Ababa , , Ethiopia	Primary health facilities , Addis Ababa , Addis Ababa , , Ethiopia
**Section Name**	**Field Name**	**Date**	**Reason**	**Old Value**	**Updated Value**
Trial Information	Target no of participants	23/04/2020	design chanage	315	279
**Section Name**	**Field Name**	**Date**	**Reason**	**Old Value**	**Updated Value**
Study Design	Intervention assignment	23/04/2020	Design change	Factorial: participants randomly allocated to either no, one, some or all interventions simultaneously	Parallel: different groups receive different interventions at same time during study
**Section Name**	**Field Name**	**Date**	**Reason**	**Old Value**	**Updated Value**
Intervention	Intervention List	23/04/2020	design change	Experimental Group, SMS text , A daily medication intake and weekly medication refill visits SMS text reminders , 4 months , Arm-1 Participants will receive a daily automated medication intake and weekly refill visit SMS text reminders in addition to standard of TB treatment with non-daily directly observed therapy. All interventions will receive the intervention at a similar time for an equal time period during the continuation phase of tuberculosis treatment., 63,	Experimental Group, SMS text , A daily medication intake and weekly medication refill visits SMS text reminders , 2 months , Arm-1 (intervention) group patients will receive a daily medication intake and weekly medication refill visit SMS text, 93,
**Section Name**	**Field Name**	**Date**	**Reason**	**Old Value**	**Updated Value**
Intervention	Intervention List	23/04/2020	design change	Experimental Group, Phone call , A daily medication intake and	

				weekly medication refill visits phone call reminders, 4 months , Arm-2 participants will receive a daily medication intake and weekly refill visit phone call reminders in addition to standard of TB treatment with non-daily directly observed therapy. All interventions will receive the intervention at a similar time for an equal time period during the continuation phase of tuberculosis treatment., 63,	
**Section Name**	**Field Name**	**Date**	**Reason**	**Old Value**	**Updated Value**
Intervention	Intervention List	23/04/2020	Design change	Experimental Group, SMS text and Phone call , A daily medication intake and weekly medication refill visits SMS text and phone call reminders , 4 months , Arm-3 participants will receive a daily medication intake and weekly refill visit SMS text and phone call reminders in addition to standard of TB treatment with non-daily directly observed therapy. All interventions will receive the intervention at a similar time for an equal time period during the continuation phase of tuberculosis treatment., 126,	Experimental Group, SMS text and Phone call , A daily medication intake and weekly medication refill visits SMS text and phone call reminders , 2 months , Arm-2 (intervention) group patients will receive a daily medication intake and weekly medication refill visit SMS text and phone call reminders., 93,
**Section Name**	**Field Name**	**Date**	**Reason**	**Old Value**	**Updated Value**
Intervention	Intervention List	23/04/2020	Design Change		Control Group, Non daily DOT alone, , 2 months , Arm-3 (control) group participants will receive the same routine standard treatment care as the intervention group, but not receive the additional intervention. Patients in the control group will be followed for an equal period as the intervention group during the continuation phase of treatment. , 93, Active-Treatment of Control Group
**Section Name**	**Field Name**	**Date**	**Reason**	**Old Value**	**Updated Value**
Outcome	OutCome List	23/04/2020	time point change	Primary Outcome, TB medication adherence , TB medication adherence at 4 and 6 months of treatment	Primary Outcome, TB medication adherence , TB medication adherence at 4 and 8 weeks of intervention
**Section Name**	**Field Name**	**Date**	**Reason**	**Old Value**	**Updated Value**
Outcome	OutCome List	23/04/2020	Design change	Secondary Outcome, TB treatment outcome, 6 months of TB treatment	Secondary Outcome, Nil, Nil
**Section Name**	**Field Name**	**Date**	**Reason**	**Old Value**	**Updated Value**
Ethics	Ethics List	23/04/2020	Ethics document uploaded	FALSE, Southern Adelaide Clinical Human Research Ethics	FALSE, Southern Adelaide Clinical Human Research Ethics

				Committee, Flinders Medical centre, Bedford Park, South Australia , Adelaide , 5042, Australia, 17 Feb 2020, , 61882046285, Health.SALHNOfficeforResearch@sa.gov.au,	Committee, Flinders Medical centre, Bedford Park, South Australia , Adelaide , 5042, Australia, 17 Feb 2020, , 61882046285, Health.SALHNOfficeforResearch@sa.gov.au, 9729_8995_4737.pdf
**Section Name**	**Field Name**	**Date**	**Reason**	**Old Value**	**Updated Value**
Ethics	Ethics List	23/04/2020	Ethics approval information updated	FALSE, Southern Adelaide Clinical Human Research Ethics Committee, Flinders Medical centre, Bedford Park, South Australia , Adelaide , 5042, Australia, 17 Feb 2020, , 61882046285, Health.SALHNOfficeforResearch@sa.gov.au, 9729_8995_4737.pdf	TRUE, Southern Adelaide Clinical Human Research Ethics Committee, Flinders Medical centre, Bedford Park, South Australia , Adelaide , 5042, Australia, 17 Feb 2020, 08 Apr 2020, 61882046285, Health.SALHNOfficeforResearch@sa.gov.au, 9729_8995_4737.pdf
**Section Name**	**Field Name**	**Date**	**Reason**	**Old Value**	**Updated Value**
Outcome	OutCome List	23/04/2020	At the random point of 4 or 8 weeks of intervention	Primary Outcome, TB medication adherence , TB medication adherence at 4 and 8 weeks of intervention	Primary Outcome, TB medication adherence , At the random point of 4 or 8 weeks of intervention
**Section Name**	**Field Name**	**Date**	**Reason**	**Old Value**	**Updated Value**
Intervention	Intervention List	18/02/2021	Study design modified	Experimental Group, SMS text , A daily medication intake and weekly medication refill visits SMS text reminders , 2 months , Arm-1 (intervention) group patients will receive a daily medication intake and weekly medication refill visit SMS text, 93,	Experimental Group, SMS text plus phone calls, A daily SMS text and weekly phone calls for medication intake and medication refill visits reminders , 2 months , Arm-1 (intervention) group participants will receive a daily SMS text and weekly phone calls for medication intake and medication refill visit reminders, 93,
**Section Name**	**Field Name**	**Date**	**Reason**	**Old Value**	**Updated Value**
Intervention	Intervention List	18/02/2021	Study design modified	Experimental Group, SMS text and Phone call , A daily medication intake and weekly medication refill visits SMS text and phone call reminders , 2 months , Arm-2 (intervention) group patients will receive a daily medication intake and weekly medication refill visit SMS text and phone call reminders., 93,	
**Section Name**	**Field Name**	**Date**	**Reason**	**Old Value**	**Updated Value**
Study Design	Masking / blinding	18/02/2021	study design modified	Outcome Assessors	Outcome Assessors, Care giver/Provider
**Section Name**	**Field Name**	**Date**	**Reason**	**Old Value**	**Updated Value**
Trial Information	Target no of participants	18/02/2021	Study design modified	279	186
**Section Name**	**Field Name**	**Date**	**Reason**	**Old Value**	**Updated Value**
Eligibility	Inclusion criteria	18/02/2021	Editorial correction	To be included into the randomized control trial (RCT) participants must be enrolled in a primary public health facility for anti-TB treatment and have attained their first	To be included into the randomized control trial (RCT) • participants must be enrolled in a primary public health facility for anti-TB treatment and have

				two months of the intensive phase of treatment, aged 18 and above years, have their own mobile phone, able to read and understand SMS text that is written in the local official language . Participants who don’t have a mobile phone will also get a chance of inclusion if they have a shared partner’s mobile phone in the household with a corroborative agreement .	attained their first two months of the intensive phase of treatment • participants must be aged 18 years and above • participants must be able to read and understand SMS text that is written in the national official language (Amharic) of Ethiopia • participants must have their own mobile phone, or • participants who do not have a mobile phone can be included if they have a shared mobile phone in the household with a collaborative agreement; (i.e. a voluntary agreement between the patient and family member living in the same household).
**Section Name**	**Field Name**	**Date**	**Reason**	**Old Value**	**Updated Value**
Eligibility	Exclusion criteria	18/02/2021	Editorial correction	Participants will be excluded from the randomized trial if they meet the following conditions: Patients whose anti-tuberculosis treatment for more than six months of treatment. Patients who unable to read and speak the national official language of Ethiopia. Patients who enrolled or agreed to enroll in another interventional study as the same time with this study.	Participants will be excluded from the randomized trial if they meet the following conditions: Patients whose anti-tuberculosis treatment prescribed for more than six months of treatment. Patients who unable to read and speak the national official language of Ethiopia. Patients who enrolled or agreed to enroll in another trial study as the same time with this study.
**Section Name**	**Field Name**	**Date**	**Reason**	**Old Value**	**Updated Value**
Contact People	Contacs List	18/02/2021	Senior researcher added to the trial		Scientific Enquiries, Paul , Arbon, Prof., Paul.arbon@flinders.edu .au, , +61418856560, Sturt Road Bedford Park , Adelaide, , Australia, Matthew Flinders Distinguished Professor
**Section Name**	**Field Name**	**Date**	**Reason**	**Old Value**	**Updated Value**
Intervention	Intervention List	18/02/2021	Editorial correction	Control Group, Non daily DOT alone, , 2 months , Arm-3 (control) group participants will receive the same routine standard treatment care as the intervention group, but not receive the additional intervention. Patients in the control group will be followed for an equal period as the intervention group during the continuation phase of treatment. , 93, Active-Treatment of Control Group	Control Group, Non daily DOT alone, , 2 months , Arm-2 (control) group participants will receive the same routine standard treatment care as the intervention group, but not receive the additional intervention. Patients in the control group will be followed for an equal period as the intervention group during the continuation phase of treatment. , 93, Active-Treatment of Control Group
**Section Name**	**Field Name**	**Date**	**Reason**	**Old Value**	**Updated Value**
Outcome	OutCome List	18/02/2021	Baseline assessment added	Primary Outcome, TB medication adherence , At the random point of 4 or 8 weeks of intervention	Primary Outcome, TB medication adherence , At the baseline, and at the random point of 4 and 8 weeks of intervention

Section Name	Field Name	Date	Reason	Old Value	Updated Value
Trial Information	Anticipated trial start date	05/03/2021	change the anticipated trial start date	01 Dec 2020	31 Mar 2021
**Section Name**	**Field Name**	**Date**	**Reason**	**Old Value**	**Updated Value**
Trial Information	Anticipated date of last follow up	05/03/2021	change the anticipated trial start date	31 May 2021	31 Jul 2021
