# Supplementary material for: Protocol of a parallel group Randomized Control Trial (RCT) for Mobile-assisted Medication Adherence Support (Ma-MAS) intervention among Tuberculosis patients
Source: PLoS One. 2021 Dec 31;16(12):e0261758. doi: 10.1371/journal.pone.0261758 (PMC8719740; doi:10.1371/journal.pone.0261758)
Supplement: Attachment — Submitted filename: Response to Reviewers.docx [file pone.0261758.s008.docx]

**Response to reviewer’s comments**

PONE-D-21-07609
Protocol of a Parallel Group Randomized Control Trial (RCT) for Mobile-assisted Medication Adherence Support (Ma-MAS) intervention among Tuberculosis Patients
PLOSONE
Dear editor and reviewers

We would like to thank you for your comments and suggestions on the protocol manuscript. We carefully reviewed your comments and suggestions one by one and revised the protocol manuscript and prepared this response.

**Editor comments**

The study is of significance and fits within the journal scope, however the manuscript needs further details to be suitable for publication.  Please see the comments below from the reviewers, in particular the drug types/scheduling details for the intervention and control groups need to be more specific.

**Response:** Thank you. We revised the protocol manuscript as per the reviewers’ comments and suggestions. We also checked the revised protocol manuscript is prepared as per the PLOS one manuscript guideline.

In addition, consider whether Figure 2. needs modifying, or whether is necessary, as at present it indicates an arrow from the intervention section to the control group, which is somewhat confusing as I am assuming the control group do not receive the intervention.

**Response:** We appreciate the editor for observing the error in Figure 2. We corrected Figure 2 in the revised protocol manuscript.

Journal Requirements:

1. When submitting your revision, we need you to address these additional requirements.

Please ensure that your manuscript meets PLOS ONE's style requirements, including those for file naming. The PLOS ONE style templates can be found at 
<https://journals.plos.org/plosone/s/file?id=wjVg/PLOSOne_formatting_sample_main_body.pdf> and 
<https://journals.plos.org/plosone/s/file?id=ba62/PLOSOne_formatting_sample_title_authors_affiliations.pdf>

**Response:** We have checked that the revised protocol manuscript is prepared based on the PLOS ONE’S style requirement.

2. We note that the grant information you provided in the ‘Funding Information’ and ‘Financial Disclosure’ sections do not match.

When you resubmit, please ensure that you provide the correct grant numbers for the awards you received for your study in the ‘Funding Information’ section

**Response:** Thank you for noted us the inconsistent statement on funding disclosure and funding information. We corrected the funding information in the revised protocol manuscript submission as this trial protocol is partially funded by Flinders University. The funder and sponsor had no role in trial design, and will not have a role in the trial implementation, analysis, and interpretation of the findings. All trial information is the responsibility of the authors.

3. In your Data Availability statement, you have not specified where the minimal data set underlying the results described in your manuscript can be found. PLOS defines a study's minimal data set as the underlying data used to reach the conclusions drawn in the manuscript and any additional data required to replicate the reported study findings in their entirety. All PLOS journals require that the minimal data set be made fully available. For more information about our data policy, please see <http://journals.plos.org/plosone/s/data-availability>.

"Upon re-submitting your revised manuscript, please upload your study’s minimal underlying data set as either Supporting Information files or to a stable, public repository and include the relevant URLs, DOIs, or accession numbers within your revised cover letter. For a list of acceptable repositories, please see <http://journals.plos.org/plosone/s/data-availability#loc-recommended-repositories>. Any potentially identifying patient information must be fully anonymized.

Important: If there are ethical or legal restrictions to sharing your data publicly, please explain these restrictions in detail. Please see our guidelines for more information on what we consider unacceptable restrictions to publicly sharing data: <http://journals.plos.org/plosone/s/data-availability#loc-unacceptable-data-access-restrictions>. Note that it is not acceptable for the authors to be the sole named individuals responsible for ensuring data access.

We will update your Data Availability statement to reflect the information you provide in your cover letter.

**Response:** Thank you for the comment. We have added a statement about data availability in the current manuscript and future trial findings. All data is included in the trial protocol manuscript, and the minimum data set of the trial findings will be available upon the study is completed and published in an international peer-reviewed open access journal.

**Response to Reviewers' comments**

1. Does the manuscript provide a valid rationale for the proposed study, with clearly identified and justified research questions?

The research question outlined is expected to address a valid academic problem or topic and contribute to the base of knowledge in the field.

Reviewer #1: Yes

Reviewer #2: Yes

2. Is the protocol technically sound and planned in a manner that will lead to a meaningful outcome and allow testing the stated hypotheses?

The manuscript should describe the methods in sufficient detail to prevent undisclosed flexibility in the experimental procedure or analysis pipeline, including sufficient outcome-neutral conditions (e.g. necessary controls, absence of floor or ceiling effects) to test the proposed hypotheses and a statistical power analysis where applicable. As there may be aspects of the methodology and analysis which can only be refined once the work is undertaken, authors should outline potential assumptions and explicitly describe what aspects of the proposed analyses, if any, are exploratory.

Reviewer #1: Partly

Reviewer #2: Yes

**Response**: We do believe that we have addressed the comments given by reviewer #1 and the revised protocol manuscript will answer this question.

3. Is the methodology feasible and described in sufficient detail to allow the work to be replicable?

Descriptions of methods and materials in the protocol should be reported in sufficient detail for another researcher to reproduce all experiments and analyses. The protocol should describe the appropriate controls, sample size calculations, and replication needed to ensure that the data are robust and reproducible.

Reviewer #1: Yes

Reviewer #2: Yes

4. Have the authors described where all data underlying the findings will be made available when the study is complete?

The [PLOS Data policy](https://journals.plos.org/plosone/s/materials-and-software-sharing) requires authors to make all data underlying the findings described in their manuscript fully available without restriction, with rare exception, at the time of publication. The data should be provided as part of the manuscript or its supporting information, or deposited to a public repository. For example, in addition to summary statistics, the data points behind means, medians and variance measures should be available. If there are restrictions on publicly sharing data—e.g. participant privacy or use of data from a third party—those must be specified.

Reviewer #1: Yes

Reviewer #2: No

**Response:** We appreciate the reviewer for correctly observe the data availability statement was not included in the first manuscript submission. We have included the data availability statement in the revised protocol manuscript and we believe the revised protocol manuscript will answer this question.

5. Is the manuscript presented in an intelligible fashion and written in Standard English?

PLOS ONE does not copyedit accepted manuscripts, so the language in submitted articles must be clear, correct, and unambiguous. Any typographical or grammatical errors should be corrected at revision, so please note any specific errors here.

Reviewer #1: Yes

Reviewer #2: Yes

6. Review Comments to the Author

1. What type of medicine is the patient taking? Is it fixed dosed combination (FDC) or combination drugs with individual strips for each drug? because if the patient is taking the last type, maybe urine tests with the measurement of the metabolite Isoniazid don't seem to be able to represent patients’ adherence because Isoniazid and Rifampicin are taken once a day while Pyrazinamide and Ethambutol are taken 3 times a day for intensive treatment phase.

**Response:** We would like to thank the reviewer for asking this question. In our RCT all participants will be treated with the same standard of care according to the national standard treatment guideline which is rifampicin, isoniazid, pyrazinamide, and ethambutol for two months followed by rifampicin and isoniazid for four months. This information is added to the revised protocol manuscript in the implementation section page 8. We designed the trial intervention to be implemented in the continuation phase of treatment in which the patient is treated with a fixed-dose combination of rifampicin and isoniazid for four months.The IsoScreen test detects isoniazid (INH) metabolites in urine. There is also evidence that showed the IsoScreen test is not influenced by demographics, smoking, alcohol and additional medication uses.

2. It will be better if the authors provide the reason or evidence why the adherence test with the urine test will be tested on the fourth and eighth weeks of intervention. Also, provide evidence that urine test has superiority to measure adherence than other methods.

**Response:** Thank you for asking why the urine test is scheduled in the fourth and eighth weeks of the intervention. The standard program for drug-sensitive TB treatment in Ethiopia is directly observed treatment (DOT) for the first two months in which patients need to come to the health facility daily followed by patients take the medication at home and come weekly for medication refill visits from 2 to 6 months of treatment. While there is evidence that showed non-adherence was high during the continuation phase of the treatment, and thus we designed the trial intervention (SMS and phone calls) at the continuation phase of the treatment. Because patients had a better adherence in the intensive phase of the treatment, we planned the urine test after the start of the intervention (SMS and phone calls) at two points which have advantage to show the trial intervention effect and utilize the test kits efficiently. Therefore, the first urine test is planned at the mid of the trial intervention which is after four weeks of SMS and phone calls, and the second test is planned at end of the trial intervention which is the eighth week. However, we will also assess adherence status at the baseline and end of intervention using the self-report method (ACTG and VAS) and clinical attendance.

Thank you for asking the urine test has superiority to measure adherence than other methods. The existing studies showed that the IsoScreen test has a high sensitivity range of 83% to 100% and high specificity of over 95%. It is a fast, relatively low cost easy to interpret, and not affected by biases which are essential for measuring the outcome correctly in our RCT. However, the adherence status is limited to recent medication use. For that reason, we planned the urine test at two different points (fourth and eighth weeks of intervention). Medication Event Monitoring System (MEMS) is one of the most accurate measures of adherence and can show long-term adherence but it is expensive in high burden TB settings like Ethiopia. While the Self-report method can show long-term adherence and is inexpensive but it lacks reliability and it can be affected by recall bias and social desirability biases. Therefore, we proposed the primary outcome adherence is to be measured by the IsoScreen test and the secondary outcome adherence is to be measured by self-report, and clinic attendance.

3. The method how to measure adherence in the abstract and the body text seems not consistent. Please make it consistent. The abstract has only been written with urine test but in the body text, there are some measurements: urine test, ACTG, VAS, and clinical attendance registration.

**Response:** The reviewer correctly noted only the urine test adherence measurement is described in the abstract. We included the self-report adherence measurements (ACTG, and VAS) clinical attendance registration in the abstract in the revised protocol manuscript.

4. How to interpret the result of the urine test? The author stated that the test result is positive when the colour changes to dark purple, or blue/ green at that time, depending on the concentration of the medication (i.e. if the medication was taken in the last 24 hours or 48 hours respectively). So if the patient was taking drugs two days ago (in the last 48 hours before the urine test), the result will be blue/green and will be categorized as positive and adherence? Shouldn't in the intensive phase of treatment the drugs have to be taken every day?

**Response:** The reviewer correctly noted the TB medications are taken daily in both the intensive and continuation phase of the treatment and the colour changes are depending on the concentration of the medication. According to the vendor-supplied user manual of the IsoScreen test, the test result is positive (adherent) when the urine colour changes to purple dark, or blue or green. Evidence also showed the urine colour changes to purple dark when the medication is taken in the last 24 hours, and blue or green when the medication is taken in the last 48 hours. Therefore, non-adherent (negative) means the medication was not taken in the last 72 hours. Therefore, we are added self-report and clinical attendance methods of adherence measurement in the proposed RCT.

5. Intervention by SMS or telephone depending on the signal and the mobile phone is on. The author mentions that if there is no response, the call will be repeated 3 times and an SMS will be sent in the morning. It is necessary to inform and advise the patient to turn on the mobile phone in the morning so that the message or call can be received by the patient.

**Response:** We agree with the reviewer's comment to clearly describe the intervention implementation. In the revised protocol manuscript, we described the information briefed to the participants such as the duration, timing and frequency of the SMS and phone calls and advice to turn on mobile phone in the morning time.

6. The authors used several methods to assess patient adherence. What if there are discrepancies in the results of the adherence examination using a urine test, Clinical Trial Group adherence questionnaire (ACTG), Visual Analogue Scales (VAS), and clinical attendance registration? For example, the result of one of the tests is categorized as non-adherence and the other tests are categorized as adherence or vice versa?

**Response:** Thank you for asking this question. Adherence measurement is difficult because there are various definitions given for it, and the existence of various methods of adherence measurement. Therefore, we planned to use the direct method of adherence measurement using detection of drug and metabolites’ concentration in urine and the indirect method using self-report (ACTG and VAS) and clinical attendance. It is recommended to use the combination of different adherence methods in the randomized control trial to see the intervention effect. This is because all method of adherence measurement has limitations and there is no gold standard method of adherence measurement. As the reviewer said we may find one patient is an adherent, for example, in urine test and non-adherent in ACTG or VAS or clinical attendance. Therefore, we have planned a separate analysis of the primary and secondary outcomes of medication adherence, measured by urine test, and self-report and clinical attendance, respectively. Indeed, we can compare the consistency of the adherence status measured by different methods.

7. Maybe authors can add Alternative analysis for student T-tests if the data is not normally distributed.

**Response:** Thank you for the comment. We can use the two sample student T-test even the data is not be normally distributed based on Moore & McCabe’s large-sample criteria which are sample 1 + sample 2 >=30. Our sample size is 186 (93 for each group)

**Reviewer #2:**

1. It is unclear which is the primary outcome of the trial, and how it will be measured. Please be more specific. Please classify only one measure as the primary outcome (e.g. compliance as measured by IsoScreen) and others as secondary (e.g. compliance as measured by self-report and clinic attendance.)

**Response:** We agree with the reviewer's comment on the primary and secondary outcomes must be clearly described. The primary outcome of the trial is adherence measured by the IsoScreen test at the fourth and eighth weeks of the intervention, and the secondary outcomes are adherence measured by self-report (ACTG and VAS) and clinical attendance. We revised the protocol manuscript as per the reviewer’s comment.

2. For the IsoScreen test, when will the test be done?

**Response:** Thank you for asking when the IsoScreen test is to be done. The urine test is to be done at the fourth and eighth weeks of the intervention. This information is included in the revised manuscript.

3. When will the ACTG adherence questionnaire be administered? Will this be considered as a binary outcome (adherent/nonadherent) or continuous (50%, 65%, etc?)

**Response:** Thank you for asking the timeline for ACTG adherence measurement and it classification. The ACTG and VAS questionnaires interview will be conducted at the baseline and end of two months of intervention (SMS and phone calls). This information is included in the outcome section on page 6. The ACTG result will be classified as adherent or non-adherent based on the response given for ACTG questions for three sections. This is described in the manuscript under outcome section page 6. Participant scores less than “somewhat satisfied” to the questions in section B and other than “never” to the questions in sections C and D are considered as non-adherence.
