# Supplementary material for: Protocol of a parallel group Randomized Control Trial (RCT) for Mobile-assisted Medication Adherence Support (Ma-MAS) intervention among Tuberculosis patients
Source: PLoS One. 2021 Dec 31;16(12):e0261758. doi: 10.1371/journal.pone.0261758 (PMC8719740; doi:10.1371/journal.pone.0261758)
Supplement: Attachment — Submitted filename: Response to reviewer.docx [file pone.0261758.s009.docx]

**Response to reviewer’s comments**

PONE-D-21-07609
Protocol of a Parallel Group Randomized Control Trial (RCT) for Mobile-assisted Medication Adherence Support (Ma-MAS) intervention among Tuberculosis Patients
PLOSONE
Dear editor and reviewers

We would like to thank you for the second time comments on the protocol manuscript. We reviewed your comments and suggestions one by one and revised the protocol manuscript.

**Editor comments**

In particular, please clarify the primary outcome and randomization definitions and timing as per the reviewer comments, and address whether a clustering effect should be allowed for in the sample size calculations.

**Response:** In the revised protocol manuscript, we make clear the description of the primary outcome according to the recommendations of the SPIRIT and CONSORT statement checklist. We also make clear the randomization and allocation statement description. We also respond to the reviewer's comment about clustering effect on the sample size calculation.

Reviewer's Responses to Questions

**Comments to the Author**

1. Does the manuscript provide a valid rationale for the proposed study, with clearly identified and justified research questions?

The research question outlined is expected to address a valid academic problem or topic and contribute to the base of knowledge in the field.

Reviewer #1: Yes

Reviewer #2: Yes

2. Is the protocol technically sound and planned in a manner that will lead to a meaningful outcome and allow testing the stated hypotheses?

The manuscript should describe the methods in sufficient detail to prevent undisclosed flexibility in the experimental procedure or analysis pipeline, including sufficient outcome-neutral conditions (e.g. necessary controls, absence of floor or ceiling effects) to test the proposed hypotheses and a statistical power analysis where applicable. As there may be aspects of the methodology and analysis which can only be refined once the work is undertaken, authors should outline potential assumptions and explicitly describe what aspects of the proposed analyses, if any, are exploratory.

Reviewer #1: Yes

Reviewer #2: Yes

3. Is the methodology feasible and described in sufficient detail to allow the work to be replicable?


Descriptions of methods and materials in the protocol should be reported in sufficient detail for another researcher to reproduce all experiments and analyses. The protocol should describe the appropriate controls, sample size calculations, and replication needed to ensure that the data are robust and reproducible.

Reviewer #1: Yes

Reviewer #2: Yes

4. Have the authors described where all data underlying the findings will be made available when the study is complete?

The [PLOS Data policy](https://journals.plos.org/plosone/s/materials-and-software-sharing) requires authors to make all data underlying the findings described in their manuscript fully available without restriction, with rare exception, at the time of publication. The data should be provided as part of the manuscript or its supporting information, or deposited to a public repository. For example, in addition to summary statistics, the data points behind means, medians and variance measures should be available. If there are restrictions on publicly sharing data—e.g. participant privacy or use of data from a third party—those must be specified.

Reviewer #1: Yes

Reviewer #2: No

**Response**: We thank you the reviwer, but we believe we have included the data availability statement in the manuscript according to the PLOS data policy.

5. Is the manuscript presented in an intelligible fashion and written in standard English?

PLOS ONE does not copyedit accepted manuscripts, so the language in submitted articles must be clear, correct, and unambiguous. Any typographical or grammatical errors should be corrected at revision, so please note any specific errors here.

Reviewer #1: Yes

Reviewer #2: Yes

6. Review Comments to the Author

Reviewer #1: The outcome of this study is only one outcome, namely adherence. This outcome will be measured by using several methods or measurement tools. Can one outcome (adherence) be a primary and secondary outcome? The authors may consider using “outcome (adherence) will be measured by direct measurement (urine test) and indirect measurement (ACTG and VAS)”, without “primary and secondary outcome”. In my opinion, using “primary and secondary outcome” but the outcome is only one (adherence) is confusing.

**Response:** We would like to thank you for the reviewer’s comment regarding the clarity of the description of the outcomes. We have reviewed the SPIRIT and consort statement checklist to decide the outcome. The CONSORT statement checklist recommended to decide the primary interest when primary outcome variable, in our case ‘adherence’, is measured at different timelines after randomization, but it is not very clear when one outcome is measured by multiple methods. While it is recommended to use the valid method of measurement for the primary end point and primary interest of analysis. Therefore, the proportion of adherence measured by the IsoScreen test at the eighth week of the intervention is the primary end point and interest of analysis. The proportion adherence measured by the IsoScreen test at the fourth week of intervention and the proportion adherence measured by self-report, and clinic appointment attendance registration will be used as the secondary interest of analysis. This correction is updated in the revised protocol manuscript document.

Reviewer #2:

1. It is unclear if the primary outcome is the ISOCreen done at 4 or 8 weeks of the intervention.

**Response:** We thank the reviewer for the comment regarding the primary outcome measured by the Isoscreen test. The reviewer correctly noted that the IsoScreen test is planned to use at the fourth and eighth weeks of the intervention (daily SMS text and weekly phone calls). In the revised protocol manuscript, we indicated our primary end point and interest of analysis is adherence measured by the IsoScreen test at the eighth week of the intervention according to the recommendations of the SPIRIT and CONSORT statement checklist. While the proportion adherence measured by the IsoScreen test at the fourth week of intervention and proportion adherence measured by self-report, and clinic appointment attendance registration will be used as the secondary interest of analysis. We have revised this statement in the outcome section of the protocol manuscript.

2. Is clustering by health facility an issue? If yes, then the sample size should account for this as should the statistical analysis. If not, then a rationale should be provided for why the outcome does not vary by the health facility.

**Response:** Thank you for inquiring about the clustering effect on the final result. In 36 primary health care facilities, we intend to conduct an individual randomized experiment. We do not believe that there will be a statistically significant outcome variance by health facility which will affect the study findings because (1) the participants will be randomized and allocated into the intervention and control groups from each health facility and (2) the average participants per each health facility is 6 which will not be sufficient to cause a statistically significant difference in outcome (adherent or non-adherent) between clusters. Moreover, all health-care facilities are identical in terms of the level of care provided, the number of health-care professionals staff, and the treatment regimens they follow.

3. Randomization: The randomization described seems to be stratified by the health facility. Please update the description accordingly. Also , is a simple randomization planned? What about blocking?

**Response:** We appreciate the reviewer's suggestion to clarify the randomization and allocation statements. Randomization and allocation of participants are planned centrally using simple randomization to allocate participants to one of two groups (intervention or control group) with an equal allocation ratio (1:1) using computer-generated algorism. Individual-level randomization is planned rather than the healthcare facility-level randomization that is applied in cluster randomized trials. In the amended manuscript, we corrected this assertion about randomization and allocation. In terms of blocking, we don't intend to apply blocking randomization in our study.

4. Data should not be cleaned in Excel - there is no trail of the corrections made and it is too easy to make other mistakes. Instead, data should be cleaned using a coding statement in STATA so that there is a clear trail of the modifications made.

**Response:** We would like to express our thanks to the reviewer for his or her input. According to the comment in the amended protocol manuscript, we corrected the data cleaning statement description.

5. All data should be available following the publication of the results.

**Response:** Thank you for this comment, but we have described the data availability statement in the manuscript according to the PLOS data policy. We are unable to fully share all the data because it has some identifiable data such as health facility name, patient’s medical record number, phone number, sex, and age which has the potential risk to identify the study participants. Thus, we said the minimum data set of the trial findings will be available upon the study is completed and published.
